# MSHHOTSA: A variant of tunicate swarm algorithm combining multi-strategy mechanism and hybrid Harris optimization

**DOI:** 10.1371/journal.pone.0290117

**Published:** 2023-08-11

**Authors:** Guangwei Liu, Zhiqing Guo, Wei Liu, Bo Cao, Senlin Chai, Chunguang Wang

**Affiliations:** 1 College of Mining, Liaoning Technical University, Fuxin, Liaoning, China; 2 College of Science, Liaoning Technical University, Fuxin, Liaoning, China; 3 School of Economics & Management, Yancheng Institute of Technology, Yancheng, Jiangsu, China; 4 China Coal Technology and Engineering Group Shenyang Research Institute, Fushun, China; 5 State Key Laboratory of Coal Mine Safety Technology, Fushun, China; Zonguldak Bülent Ecevit University: Zonguldak Bulent Ecevit Universitesi, TURKEY

## Abstract

This paper proposes a novel hybrid algorithm, named Multi-Strategy Hybrid Harris Hawks Tunicate Swarm Optimization Algorithm (MSHHOTSA). The primary objective of MSHHOTSA is to address the limitations of the tunicate swarm algorithm, which include slow optimization speed, low accuracy, and premature convergence when dealing with complex problems. Firstly, inspired by the idea of the neighborhood and thermal distribution map, the hyperbolic tangent domain is introduced to modify the position of new tunicate individuals, which can not only effectively enhance the convergence performance of the algorithm but also ensure that the data generated between the unknown parameters and the old parameters have a similar distribution. Secondly, the nonlinear convergence factor is constructed to replace the original random factor *c*_1_ to coordinate the algorithm’s local exploitation and global exploration performance, which effectively improves the ability of the algorithm to escape extreme values and fast convergence. Finally, the swarm update mechanism of the HHO algorithm is introduced into the position update of the TSA algorithm, which further balances the local exploitation and global exploration performance of the MSHHOTSA. The proposed algorithm was evaluated on eight standard benchmark functions, CEC2019 benchmark functions, four engineering design problems, and a PID parameter optimization problem. It was compared with seven recently proposed metaheuristic algorithms, including HHO and TSA. The results were analyzed and discussed using statistical indicators such as mean, standard deviation, Wilcoxon’s rank sum test, and average running time. Experimental results demonstrate that the improved algorithm (MSHHOTSA) exhibits higher local convergence, global exploration, robustness, and universality than BOA, GWO, MVO, HHO, TSA, ASO, and WOA algorithms under the same experimental conditions.

## 1. Introduction

With science and technology’s continuous development and progress, we face increasingly complex engineering problems characterized by non-convexity, multimodality, and high-dimensional variability [1, 2]. The complexity of these problems renders traditional optimization methods based on gradient descent ineffective [3]. However, solving these intricate engineering problems is crucial to advancing national development and promoting scientific and technological advancements. Therefore, it is imperative to conduct in-depth research and explore the development of novel optimization algorithms and techniques.

In recent years, many scholars have proposed a series of metaheuristic algorithms based on biological evolution, animal social behavior, and natural physical phenomena to address these complex engineering constraint problems. For example, Genetic Algorithm (GA) [4, 5], Ant Colony Optimization (ACO) [6], Artificial Rabbits Optimization (ARO) [7], Grey Wolf Optimizer (GWO) [8], Grasshopper Optimization Algorithm (GOA) [9], Multi-verse Optimizer (MVO) [10], Butterfly Optimization Algorithm (BOA) [11], Harris Hawks Optimization (HHO) [12], Atom search optimization (ASO) [13], Whale Optimization Algorithm (WOA) [14], among others.

These metaheuristic algorithms solve complex engineering constraints by simulating natural organisms’ population behavior and survival hunting strategies. They do not rely on the specific form and characteristics of the problem, making them applicable to various complex engineering problems. The strengths of these algorithms lie in their broad applicability and the fact that they do not require specific formalization or prior knowledge about the situation. As a result, they can achieve excellent optimization results for a wide range of practical complex engineering problems. For instance, scholars like Altan have applied various metaheuristic algorithms to address challenges in UAV path planning and tracking [15, 16], real-time detection of agricultural plant diseases [17], cryptocurrency forecasting [18], crude oil time series prediction [19, 20], and wind speed prediction [21]. Additionally, GA and its variants have been employed in production scheduling [22, 23], allocation optimization [24], search and localization tasks [25, 26], and neural network optimization [27–29]. Furthermore, other metaheuristic algorithms, such as ASO, GWO, WOA, HHO, and their variations, have also been widely applied to address complex optimization problems in fields such as battery modeling [30, 31], feature selection [1, 32–37] and path optimization [38–41].

Although these metaheuristic algorithms have been applied to solve complex engineering constraint problems, according to the No Free Lunch Theorem, no single optimization algorithm can be universally effective for solving all optimization problems [42]. Therefore, when dealing with complex engineering constraint problems, selecting an appropriate optimization algorithm is crucial. Scholars such as Kaur S have proposed a Tunicate Swarm Algorithm (TSA) [43], which simulates the hunting and population behavior of tunicate swarms and applies it to solve various engineering constraints. For example, in image segmentation, Houssein and other scholars combined TSA with the local escaping operator (LEO) to propose the TSA-LEO algorithm, which was successfully applied to image segmentation [44]. Awari and colleagues integrated deep learning methods with the TSA algorithm and successfully applied them to three-dimensional dental image segmentation and classification [45]. Akdağ improved tunicates’ updating strategy and collective behavior in engineering optimization, proposing the Modified Tunicate Swarm Algorithm (M-TSA), which achieved successful applications in multiple engineering optimization problems [46]. Similarly, Rizk-Allah and others introduced the Enhanced Tunicate Swarm Algorithm (ETSA) to solve large-scale nonlinear optimization problems [47]. Besides, the TSA algorithm and its various variants have also received extensive attention in economic scheduling [48], distribution network optimization [49, 50], disease prediction [51, 52], and other domains. The TSA algorithm and its variants have shown significant effectiveness in solving complex engineering problems, demonstrating their potential advantages and applications. These excellent improvement mechanisms can be mainly categorized into three types:

(1) Population Diversity Enhancement Mechanism

For metaheuristic algorithms, the quality of optimization results is often closely related to population diversity. The richer the population diversity, the more likely the algorithm can escape from local optima and approach the global optimum through iterative optimization. Therefore, in the improvement mechanisms of the TSA algorithm, enhancing population diversity remains one of the main methods to improve its convergence and optimization performance. Among them, the typical approaches include chaotic initialization and reverse learning mechanisms. For example, Tent mapping [53, 54] and Halton sequence [55] have been used to enhance the population diversity of TSA with good optimization performance; meanwhile, Abhishek Sharma [56], Essam H. Houssein [57] and Jianzhou Wang [58] have applied the backward learning mechanism to TSA algorithm, which not only enhances the This mechanism not only enhances the population diversity but also improves the convergence performance of the TSA algorithm.

(2) Swarm Behavior and Individual Position Update Mechanism

In TSA, tunicate individuals’ position updates and swarm behaviors’ selection play a crucial role in the algorithm’s global exploration and convergence speed. To address this, researchers have employed nonlinear parameter adjustment mechanisms and dynamic adaptive position update mechanisms to improve the optimization performance of TSA. For instance, In terms of nonlinear parameter correction, researchers have used nonlinear functions such as exponential functions [58], Levy flight functions [53, 54, 59], cosine functions [55] and simple polynomial functions [60] instead of linear functions in TSA, which significantly improve the convergence performance of TSA in search of the best. As for dynamic adaptive position updating, Rizk-Allah et al. effectively enhanced the convergence speed of the algorithm by introducing an ingestion parameter to dynamically adjust the search step of the tunicate individual [47]. ARABALI et al. proposed an adaptive TSA algorithm (ATSA) by changing the search between the candidate and optimal solutions of the tunicate swarm. They applied it successfully to expand the base optimization [61]. Besides, Lévy distribution [62], Cauchy distribution [62], Gaussian distribution [62], adaptive competitive window [63], adaptive distribution [64] and adaptive parameters [44] are also used for the dynamic adaptive update of the tunicate position.

(3) Multi-Algorithm Fusion Mechanism

By integrating different metaheuristic algorithms, we can effectively leverage their respective strengths and mitigate their weaknesses, thereby improving the overall optimization performance of the fused algorithms. For example, HOUSSEIN et al. proposed a TSA-LEO algorithm by fusing TSA with a local escape operator (LEO) to enhance TSA’s convergence speed and local search performance. They applied it successfully to solve the image segmentation problem [44]. Chouhan et al. fused TSA with the GWO algorithm and successfully solved the multi-path routing protocol problem [65]. Doraiswami et al. fused TSA with the Jaya algorithm and used it for the GAN network, proposed Jaya-TSA based GAN prediction method, and used it successfully for patient prediction of COVID-19 [66]. In addition, the fusion of TSA with the BOA algorithm [67] and TSA with the FPA algorithm [68] can also effectively enhance the superior performance of each other, indicating that the combination of the respective search advantages of different algorithms can effectively balance the global exploration and local exploitation capabilities of the algorithms.

The above studies have strengths, as they have significantly improved and applied to the TSA algorithm in different periods and domains. These improvements mainly focus on population initialization, nonlinear convergence factor, and hybrid algorithms. Building on these improvement strategies, we propose a hybrid algorithm known as the Multi-Strategy Hybrid Harris Hawks Tunicate Swarm Optimization Algorithm (MSHHOTSA). Specifically, the main contributions of this paper are as follows:

1. Inspired by neighborhood thinking and the principles of thermographic distribution, we developed a hyperbolic tangent domain and nonlinear fast convergence factors, which are used to update the population positions and behaviors in the TSA algorithm. These new update strategies help improve the algorithm’s convergence accuracy and better balance global exploration and local exploitation abilities.
2. Inspired by hybrid algorithm mechanisms, we introduce the population behavior of the Harris Hawks Optimization (HHO) algorithm into the TSA algorithm to enhance the population diversity of TSA. By introducing new behavioral strategies, the algorithm can better explore the search space and thus have a higher chance of finding global optima.
3. We integrate the hyperbolic tangent domain, nonlinear fast convergence factor, and hybrid mechanisms to propose the Multi-Strategy Hybrid Harris Hawks Tunicate Swarm Optimization Algorithm (MSHHOTSA). By synergistically utilizing the advantages of different strategies, we further improve the algorithm’s optimization performance and robustness.
4. The MSHHOTSA algorithm is applied to 18 benchmark functions and five engineering optimization problems with constraints. Experimental results demonstrate that our algorithm exhibits significant performance when solving these problems.
5. We conduct an in-depth analysis of the shortcomings of the MSHHOTSA algorithm, providing a reference for improving or proposing new metaheuristic algorithms.

The main structure of this article is as follows: In Section 2, the standard Tunicate Swarm Optimization algorithm is introduced, including its principles and basic process. In Section 3, the proposed MSHHOTSA algorithm is described. Specifically, Subsection 3.1.1 introduces the construction process of the hyperbolic tangent domain, and Subsection 3.1.2 describes the construction process of the nonlinear fast convergence factor. Section 3.2 discusses the hybrid approach of the Harris Hawks Optimization (HHO) algorithm and the Tunicate Swarm Optimization (TSA) algorithm. Furthermore, Section 3.4 analyzes the complexity of the proposed MSHHOTSA algorithm. In Section 4, through multiple sets of experiments, the advantages and disadvantages of the MSHHOTSA algorithm are thoroughly researched and discussed. In Section 5, we apply the MSHHOTSA algorithm to solve five complex engineering optimization problems. Finally, Section 6 summarizes the current work and outlines potential future research directions.

## 2. Tunicate swarm algorithm (TSA)

The tunicate swarm algorithm is a metaheuristic algorithm proposed by Kaur S et al. [43], inspired by the swarm foraging behavior of tunicate animals in the ocean, which simulates the jet propulsion and swarm behavior of tunicate animals in the foraging process. The algorithm focuses on finding the optimal solution in the solution space by the following four conditions.

**(1) Avoid conflicts between tunicate individuals**

In order to avoid conflicts among tunicate individuals, the individual position of the new tunicate is calculated using $\vec{A}$, that is,

$$\vec{A}=\frac{\vec{G}}{\vec{M}}$$ (1)

$$\vec{G}=c_{2}+c_{3}-\vec{F}$$ (2)

$$\vec{F}=2c_{1}$$ (3)

$$\vec{M}=\lfloor P_{\min}+c_{1}(P_{\max}-P_{\min})\rfloor$$ (4)

Where $\vec{A}$ is the position of the new tunicate individual; $\vec{G}$ is the gravitational force; $\vec{M}$ is the social force between tunicate individuals; $\vec{F}$ is the deep-sea current advection; *P*_min_ = 1 and *P*_max_ = 4 represent the initial velocity and auxiliary velocity of the tunicate individual for social interaction, respectively; variables *c*_1_, *c*_2_ and *c*_3_ are random numbers between [0,1].

**(2) Move to the optimal neighbor**

After avoiding the search conflict among tunicate individuals, the tunicate individuals in the tunicate population all move to the optimal neighbor, and this behavior can be expressed as:

$$\vec{PD}=|\vec{FS}-rand\cdot \vec{p_{p}(x)}|$$ (5)

Where $\vec{PD}$ denotes the distance between the food source and the tunicate individual, $\vec{FS}$ is the location of the food source, $\vec{P_{P}(x)}$ is the current location of the tunicate individual, *x* is the number of current iterations, *rand*∈[0,1].

**(3) Converge to the position of the optimal tunicate individual**

When the above conditions are satisfied, each tunicate individual eventually approaches the optimal position, and the process is described as:

$$\vec{P_{P}(x^{*})}=\{\begin{array}{l} \vec{FS}+\vec{A}\cdot \vec{PD},\mathrm{if}\;rand\geq 0.5\\ \vec{FS}-\vec{A}\cdot \vec{PD},\mathrm{if}\;rand<0.5 \end{array}$$ (6)

Where $\vec{P_{P}(x^{*})}$ represents the updated tunicate individual position.

**(4) Swarm Behavior of Tunicates**

The tunicate swarm algorithm first saves the first two optimal solutions, and then updates the remaining tunicate individual positions according to the best tunicate individual positions, that is:

$$\vec{P_{P}(x+1)}=\frac{\vec{P_{P}(x)}+\vec{P_{P}(x+1)}}{2+c_{1}}$$ (7)

**Fig 1:**
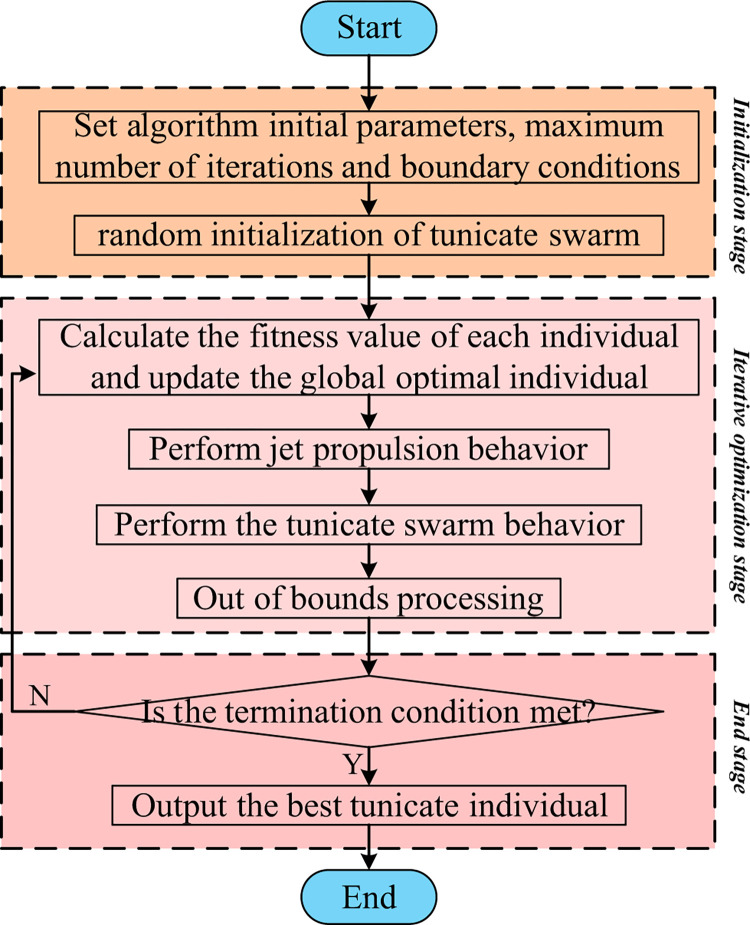
Flowchart of TSA [43].

In summary, the specific flow of the TSA algorithm can be obtained as shown in Fig 1.

### 3. The MSHHOTSA algorithm

In the Tunicate Swarm Algorithm (TSA), jet propulsion behavior primarily serves to approximate the optimal value in the solution space rapidly. In contrast, swarm behavior aims to ensure quick convergence of the algorithm and enhance its global exploration capability. To enhance TSA’s optimization performance in the solution space, this paper proposes two improvements: updating tunicate individuals’ position parameters and updating their swarm behavior.

### 3.1 Update the position parameters of the tunicate individual

#### 3.1.1 Hyperbolic tangent domain modification parameters

From Eq (6), it is evident that the position of the new tunicate animal (parameter $\vec{A}$) generated by the TSA algorithm during jet propulsion behavior determines the location of the following tunicate individual. The algorithm can be considered to have converged to the optimal solution in the solution space when there is no significant change in the position of consecutive tunicate individuals. In conjunction with Eq (1), parameter $\vec{A}$ primarily wanders randomly within a space defined by three random numbers. A larger magnitude of $\left|\vec{A}\right|$ indicates that the current tunicate individual is farther from the current optimal tunicate, increasing the probability of finding a better individual and enhancing TSA’s global exploration performance. Conversely, as $|\vec{A}|\to 0$, the current tunicate individual approaches the current optimal tunicate, accelerating the discovery of a better individual and increasing TSA’s local exploitation performance.

**Fig 2:**
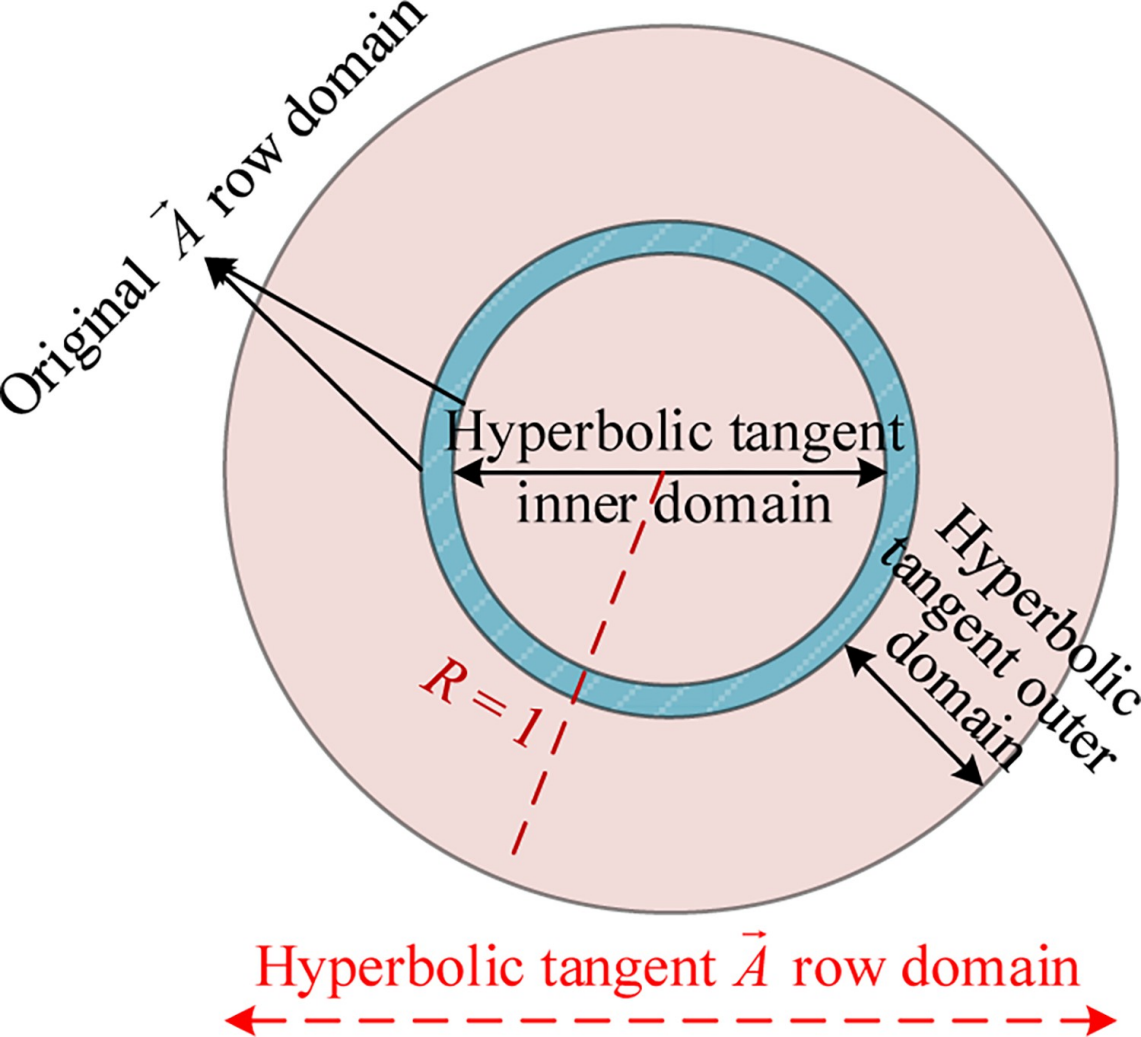
Hyperbolic tangent $\vec{A}$ row domain.

Therefore, to improve the superior performance of TSA, we drew inspiration from the neighborhood and heat map distribution concepts. The aim is to increase the value of the parameter $\left|\vec{A}\right|$ while biasing the distribution of $\vec{A}$ towards 0. This approach enhances the algorithm’s global exploration capability while accelerating its convergence speed. In this study, we introduced the hyperbolic tangent function to construct a hyperbolic tangent $\vec{A}$ row domain based on the hyperbolic tangent function (see Fig 2 for details).

In Fig 2, the blue-green region represents the random traversal space of the original parameter $\vec{A}$, referred to as the original $\vec{A}$ row domain. The light red region represents the perturbation space added by the hyperbolic tangent function, known as the hyperbolic tangent domain. The random traversal space of the modified parameter $\vec{A}$ is the combination of the hyperbolic tangent domain and the original $\vec{A}$ row domain, forming the hyperbolic tangent $\vec{A}$ row domain. The mathematical model of the hyperbolic tangent $\vec{A}$ row domain constructed based on Fig 2 is expressed as follows:

$$\vec{A}=\frac{\vec{G}}{\vec{M}}+2Thd(x)$$ (8)

where, in order to ensure that the perturbation of the hyperbolic tangent function to the parameter $\vec{A}$ remains within the unit circle, let

$$Thd(x)=\{\begin{array}{l} -\tanh(x)\;r\leq 0.5\\ \tanh(x)\;r>0.5 \end{array}$$ (9)

where *Thd*(1) = 0.5, and *x* is the number of current iterations, *r* is a random number between [0,1], *tanh*(*x*) is a hyperbolic tangent function, and the equation is:

$$\tanh(x)=\frac{e^{x}-e^{-x}}{e^{x}+e^{-x}}$$ (10)

**Fig 3:**
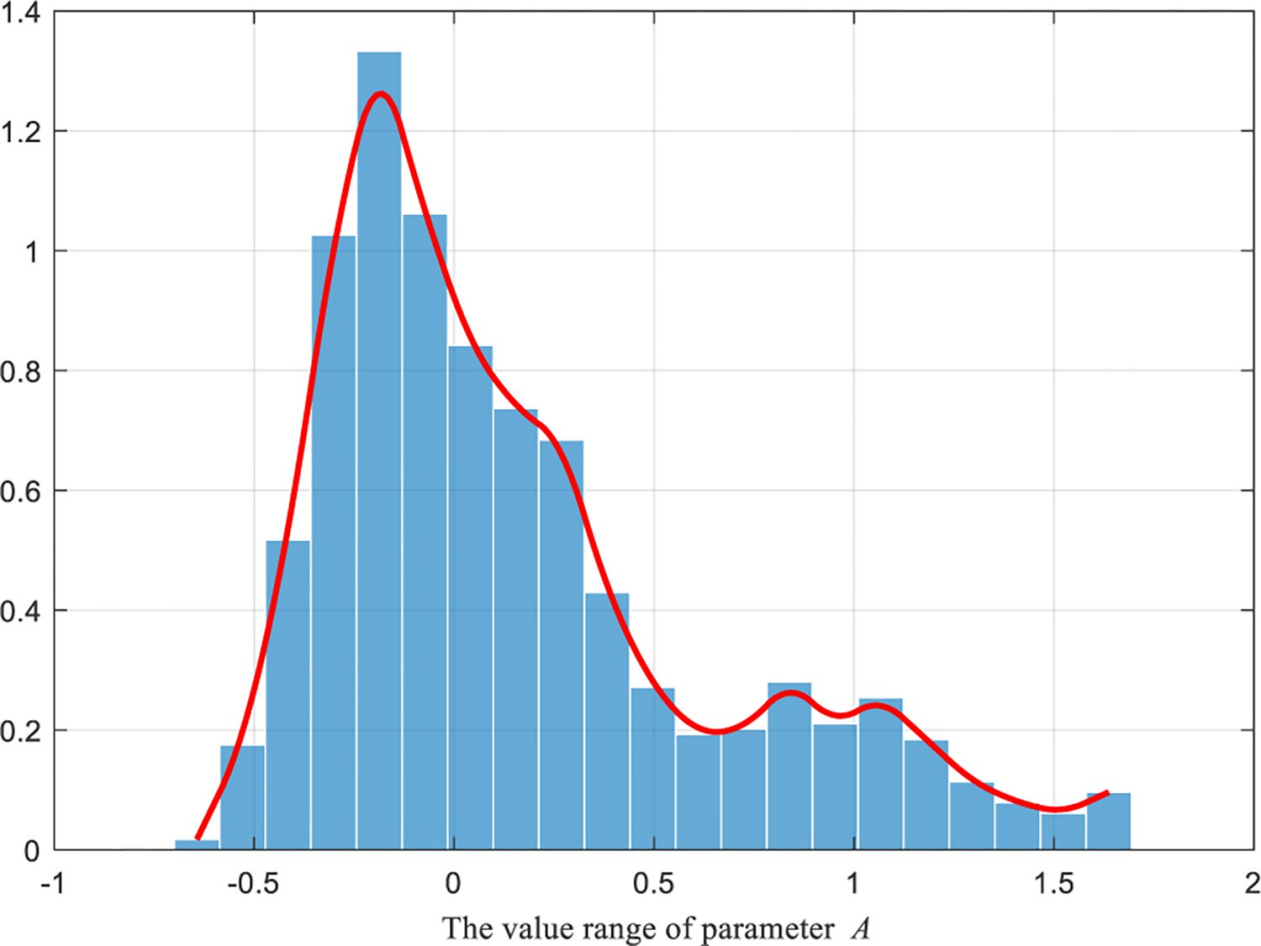
Numerical distribution of parameter $\vec{A}$ without parameter perturbation.

**Fig 4:**
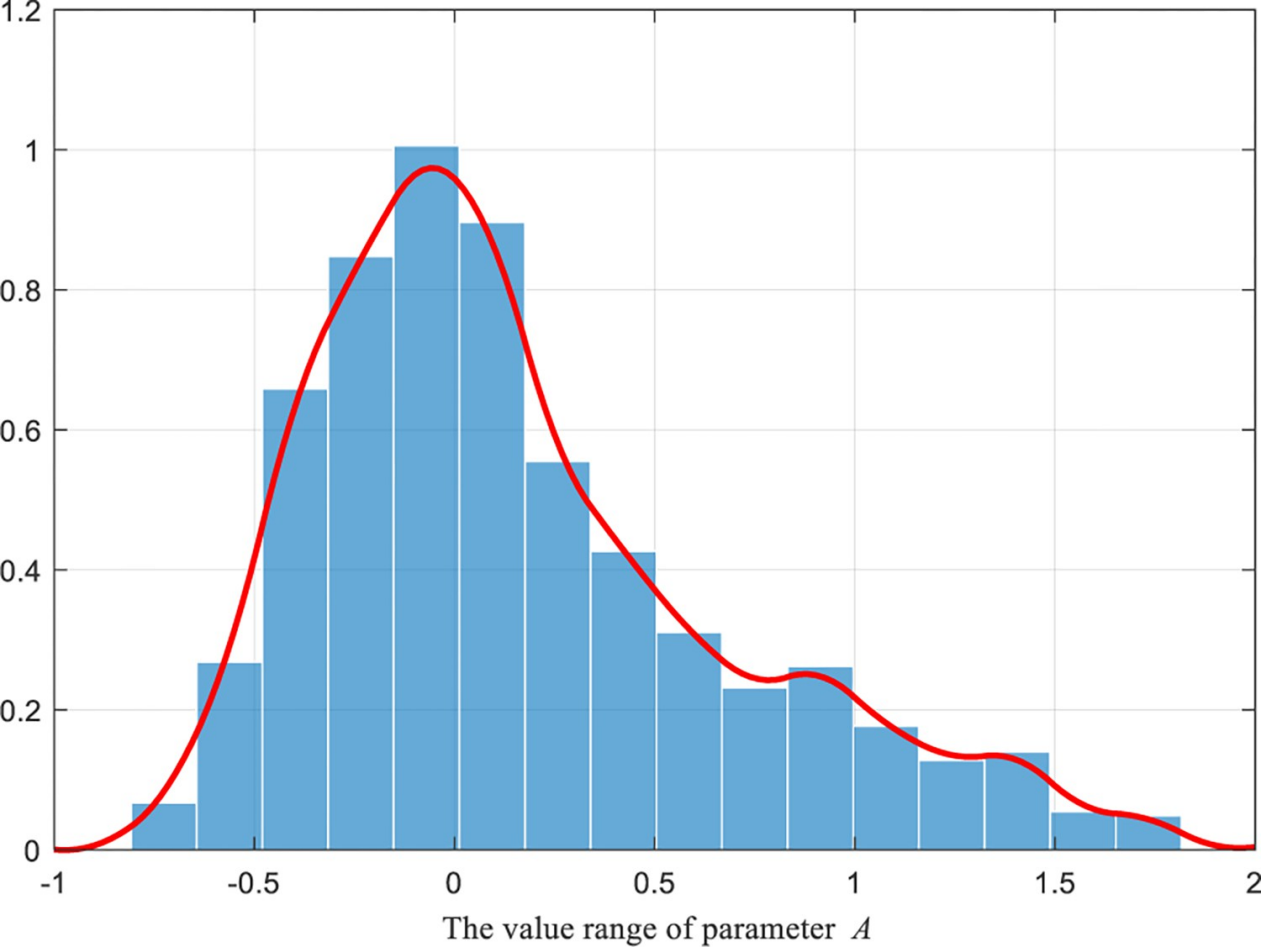
Added the numerical distribution of the parameter $\vec{A}$ perturbed in the hyperbolic tangent domain.

To sum up, according to Eq 8–Eq 10, the numerical distribution images of the original $\vec{A}$ row domain and the hyperbolic tangent $\vec{A}$ row domain are respectively generated under 1000 iterations, as shown in Figs 3 and 4.

From the comparison between Figs 3 and 4, significant changes in the data distribution of parameter $\vec{A}$ can be observed with and without the hyperbolic tangent domain perturbation. Firstly, the data distribution of parameter $\vec{A}$ transformed from a spiky truncated tail to a rounded trailing bottom. This shift indicates that the interval range (i.e., random wandering range) of parameter $\vec{A}$ expanded and became more concentrated around the 0 intervals after the perturbation. As a result, the tunicate individuals were not only encouraged to explore better values in a larger space (due to the increased interval range) but also were able to approach the optimal neighbors more rapidly (due to the data concentration around the 0 intervals). Secondly, despite the changes in the parameter data distribution after introducing perturbation, the overall distribution still exhibited similarity, ensuring the uniformity of data information effectively.

In summary, the modified hyperbolic tangent $\vec{A}$ row domain not only effectively preserves the similarity in data distribution with the original $\vec{A}$ row domain but also allows for adjusting the random walking space. This adjustment effectively balances the algorithm’s global exploration and local exploitation capabilities.

#### 3.1.2 Nonlinear convergence factor update mechanism for tunicate swarm behavior

**Fig 5:**
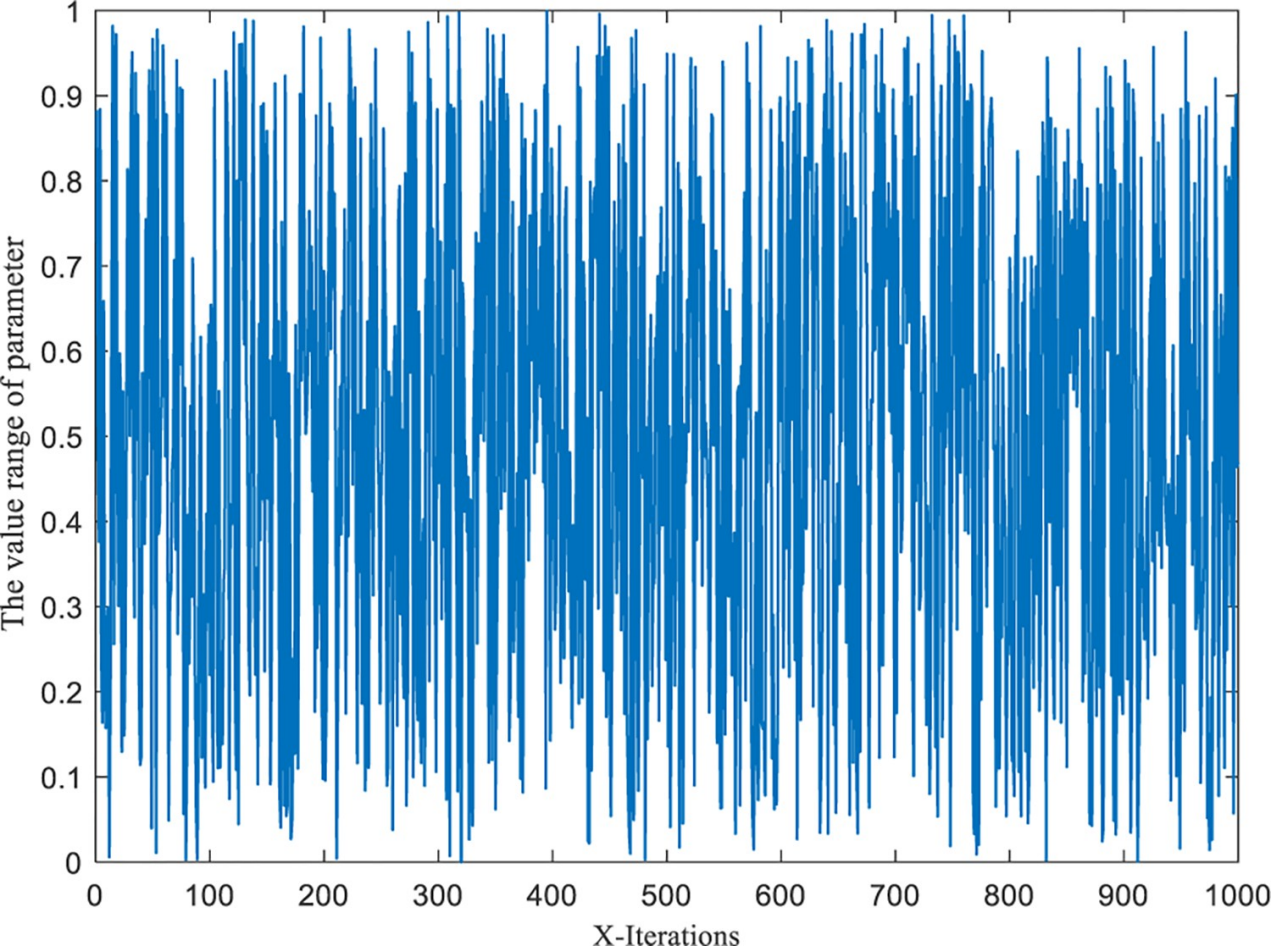
Random parameter factor.

In TSA, it is observed from the population behavior of the tunicate organisms (Eq (7)) that the location of the best tunicate individual is influenced by its place and the location of the previous tunicate individual. The algorithm reaches the extreme value when the site of the last tunicate individual is the same as the best tunicate location. Therefore, in Eq (7), the random parameter *c*_1_ plays a crucial role in determining whether the algorithm can escape local optimal solutions and reach global optimal solutions. However, the randomness of the parameter *c*_1_ during the continuous iterations of the algorithm (see Fig 5) significantly impacts the algorithm’s convergence, leading to an imbalance between global exploration and local exploitation. To enhance the TSA search performance more effectively, this paper introduces a deterministic nonlinear convergence factor to replace the random parameter *c*_1_ in Eq (7) (Eq (3) remains unchanged). The modified Eq (7) is expressed as:

$$\vec{P_{P}(x+1)}=\frac{\vec{P_{P}(x)}+\vec{P_{P}(x+1)}}{2+w(x)}$$ (11)

where, the equation of the introduced nonlinear convergence factor is:

$$w(x)=\cos(\frac{2\pi x}{x_{\max}})-\sin(\frac{\pi x}{x_{\max}})-\log(\frac{x}{2})$$ (12)

**Fig 6:**
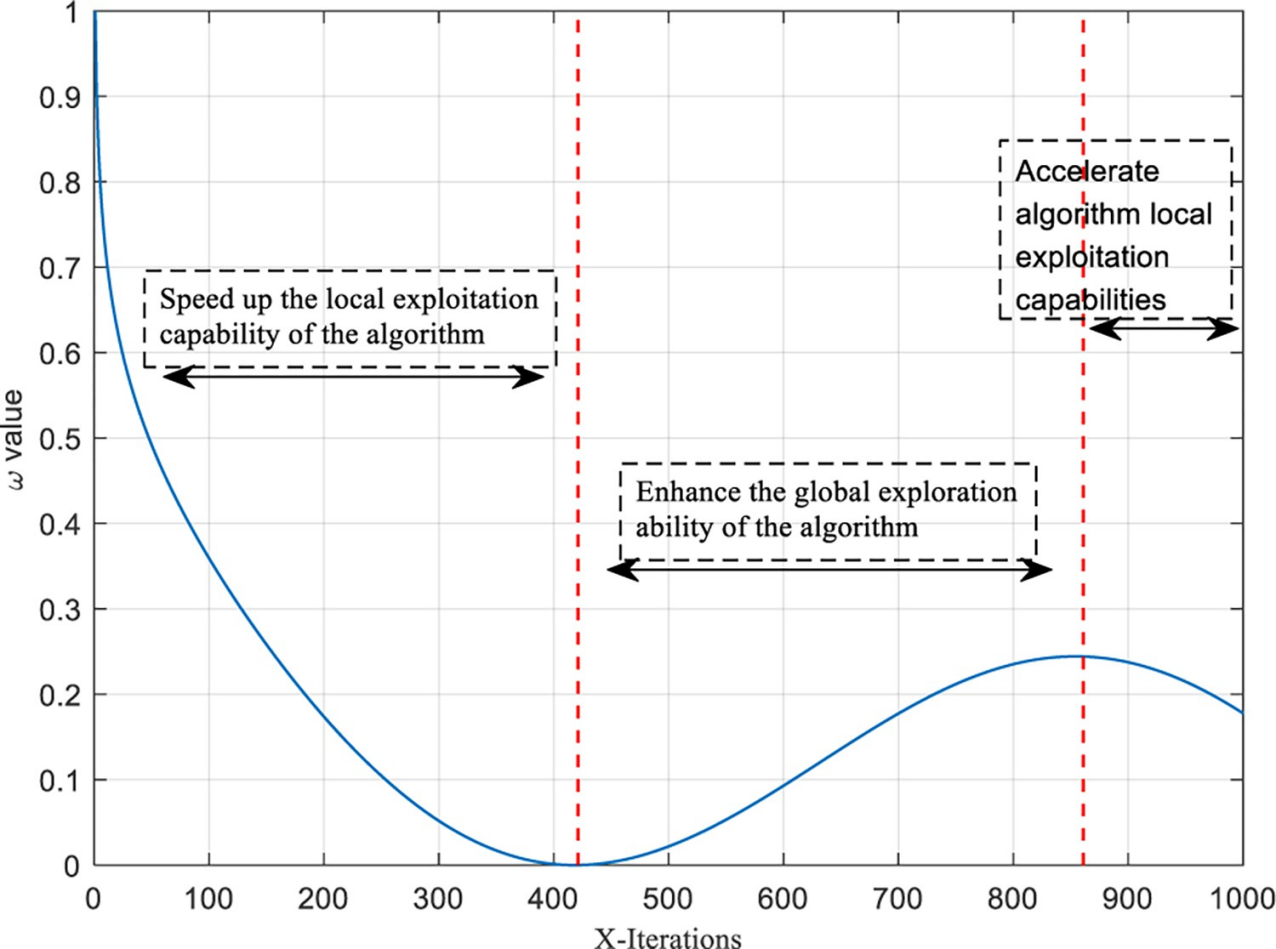
Nonlinear fast convergence factor.

Where *x* represents the current number of iterations, and *x*_max_ is the maximum number of iterations. In Eq (12), sine and cosine functions control the algorithm’s fluctuation during iterations, crucially contributing to the algorithm’s global exploration performance. The negative logarithm function ensures a systematic decrease in the algorithm as iterations progress, which is pivotal in enhancing the algorithm’s local exploitation performance. To ensure that the convergence factor and the interval range of the random factor *c*_1_ are identical, *w*(*x*) is normalized. The corresponding function expression and graph are illustrated in Eq (13) and Fig 6, respectively.


$$\omega (x)=\frac{w(x)-w_{\min}}{w_{\max}-w_{\min}}$$ (13)

So, Eq (11) can be redefined as:

$$\vec{P_{P}(x+1)}=\frac{\vec{P_{P}(x)}+\vec{P_{P}(x+1)}}{2+\omega (x)}$$ (14)

After the introduction of the nonlinear fast convergence factor, the TSA iterative process can be divided into three stages: the pre-iterative stage, the mid-iterative stage, and the post-iterative stage by analyzing Eq (14) and Fig 6.

**3.1.2.1 Pre-iterative stage: Accelerate the convergence speed of the algorithm.** At this time, the nonlinear convergence factor *ω*(*x*) rapidly approaches 0, and the $\vec{P_{P}(x)}$ also rapidly approaches $\vec{P_{P}(x+1)}$, that is, the algorithm rapidly approaches the current optimal solution in the solution space.

**3.1.2.2 Mid-iterative stage: Enhance the ability of the algorithm to escape the local extremum.** The relationship between $\vec{P_{P}(x)}$ and $\vec{P_{P}(x+1)}$ becomes more and more distant as *ω*(*x*) changes from fast approaching 0 to slowly increasing. This process represents the process by which the algorithm jumps out of the local extremum to try to find if there is a better value in the solution space.

**3.1.2.3 Post-iterative stage: Improve the convergence speed of the algorithm.** At this time, *ω*(*x*) changes from gradually increasing to slowly decreasing, then $\vec{P_{P}(x)}$ gradually approximates $\vec{P_{P}(x+1)}$. This process represents the process of the algorithm approaching the optimal solution again after jumping out of the local extremes.

In summary, introducing the deterministic nonlinear convergence factor instead of the random factor addresses the uncertainty impact of randomness on the algorithm’s convergence. It also achieves a more effective balance between global exploration and local exploitation capabilities to a certain extent.

### 3.2 Tunicate swarm algorithm based on harris hawks optimization

In Section 3.1, we introduce the hyperbolic tangent domain and the nonlinear fast convergence factor to modify the jet propulsion and swarm behavior of TSA, respectively. These modifications somewhat improve TSA’s global exploration and local exploitation abilities. However, upon considering the overall iterative process of the algorithm, we observe that these two improvement mechanisms mainly promote the local exploitation performance of the algorithm. To better balance the algorithm’s global exploration and local exploitation capabilities, we introduce the Harris Hawks Optimization to enhance TSA’s superior performance.

Harris hawks optimization is a meta-heuristic algorithm that simulates the collaborative behavior of a swarm of harris hawks during hunting. The process of solving complex problems using this algorithm can be divided into two stages: exploration and exploitation.

**(1) Exploration stage (|*E*|≥1)**

When |*E*|≥1, the Harris hawk randomly inhabits in the solution space and waits for an opportunity to observe the prey. Under the perching strategy with equal probability 0.5, the mathematical model of the Harris hawk hunting behavior is:

$$\vec{P_{P}(x+1)}=\{\begin{array}{l} \vec{P_{\mathrm{rand}}(x)}-r_{1}|\vec{P_{\mathrm{rand}}(x)}-2r_{2}\vec{P_{P}(x)}|,\;q\geq 0.5\\ (\vec{P_{\mathrm{rabbiit}}(x)}-\vec{P_{m}(x)})-r_{3}(\mathrm{lb}+r_{4}(\mathrm{ub}-\mathrm{lb})),\;q<0.5 \end{array}$$ (15)

Where $\vec{P_{\mathrm{rabbit}}(x)}$ is the position of the prey rabbit at the *x*-th iteration; $\vec{P_{m}(x)}$ is the center position of the Harris hawk flock at the *x*-th iteration; $\vec{P_{\mathrm{rand}}(x)}$ is the random position of the Harris hawk at the *x*-th iteration; *r* and *q* are random numbers between [0,1], and lb and ub are the upper and lower bounds for solving the problem; *E* is the prey escape energy, and its equation is:

$$E=2E_{0}(1-x/x_{\max})$$ (16)

Where *x* is the current number of iterations; *x*_max_ is the maximum number of iterations of the algorithm; *E*_0_ is a random number between [–1,1], indicating the initialized energy value.

**(2) Exploitation stage (|*E*|<1)**

When |*E*|<1, HHO implements four strategies of ‘soft encirclement’, ‘hard encirclement’, ‘fast dive soft encirclement’ and ‘fast dive hard encirclemen’ through random numbers to realize the Predation of prey rabbit.

**1) Soft encirclement**

When |*E*|≥0.5 and the random number is greater than or equal to 0.5, the Harris hawk executes the soft encirclement strategy, and its mathematical model is:

$$\vec{P_{P}(x+1)}=\vec{P_{r}(x)}-\vec{P_{P}(x)}-E|\vec{BS}|$$ (17)

Where $\vec{P_{r}(x)}=\vec{P_{\mathrm{rabbit}}(x)}$, $\vec{BS}=J\times \vec{P_{\mathrm{rabbit}}(x)}-\vec{P_{P}(x)}$; *J* is a random number between [0,2], indicating the jumping ability of prey rabbits.

**2) Hard encirclement**

When |*E*|<0.5 and the random number is greater than or equal to 0.5, Harris hawk executes a hard encirclement strategy, whose mathematical model is:

$$\vec{P_{P}(x+1)}=\vec{P_{r}(x)}-E|\vec{P_{r}(x)}-\vec{P_{P}(x)}|$$ (18)

Where $\vec{P_{r}(x)}=\vec{P_{\mathrm{rabbit}}(x)}$.

**3) Fast dive soft encirclement**

When |*E*|≥0.5 and the random number is less than 0.5, that is, Harris Hawk raid failed, the algorithm executes random walk $\vec{Z}$; that is, to perform a fast dive soft encirclement strategy whose mathematical model is expressed as:

$$\vec{P_{P}(x+1)}=\vec{P_{\mathrm{rabbit}}(x)}-E|\vec{BS}|$$ (19)

$$\vec{Z}=\vec{P_{P}(x+1)}+\vec{S}\times \mathrm{LF}(D)$$ (20)

Where $\vec{BS}=J\times \vec{P_{\mathrm{rabbit}}(x)}-\vec{P_{P}(x)}$; *J* is a random number between [0,2]; $\vec{S}$ is a random vector; *D* is the problem dimension; LF is the Levy flight function.

**4) Fast dive hard encirclement**

When |*E*|<0.5 and the random number is less than 0.5, the Harris hawk performs a fast dive hard encirclement strategy whose mathematical model is expressed as:

$$\vec{P_{P}(x+1)}=\vec{P_{\mathrm{rabbit}}(x)}-E|\vec{BM}|$$ (21)

Where $\vec{BM}=J\times \vec{P_{\mathrm{rabbit}}(x)}-\vec{P_{m}(x)}$; *J* is a random number between [0,2].

To enhance the tunicate individual’s ability to escape local optima while maintaining improved convergence performance, the TSA algorithm incorporates the HHO algorithm. After the TSA algorithm obtains the optimal tunicate position through the improved strategy in Section 3.1, the HHO algorithm is introduced to further update the tunicate position, resulting in a better individual place. With the fusion of HHO and TSA algorithms, the MSHHOTSA performs a hybrid search optimization process that consists of two stages: exploration and exploitation.

1. During the exploration stage, i.e., when |*E*|≥1, the TSA algorithm updates the optimal tunicate position. This stage primarily facilitates the optimization process of the TSA algorithm itself.
2. In the exploitation stage, i.e., when |*E*|<1, the HHO algorithm updates the optimal tunicate position. Within this stage, the four strategies of the HHO algorithm are mainly employed to enhance the process of the current tunicate individual approaching the optimal tunicate individual.

**Fig 7:**
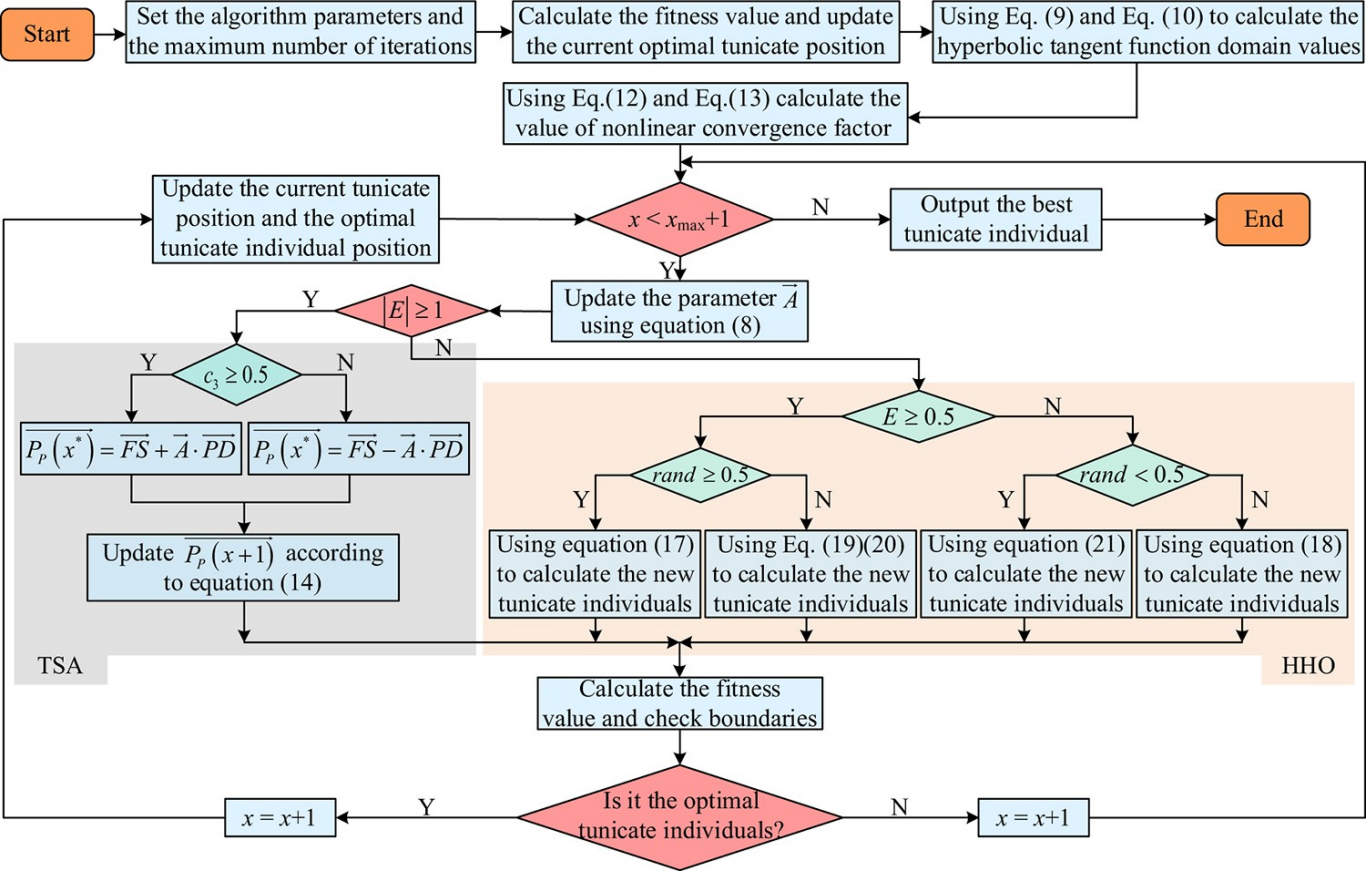
Flowchart of MSHHOTSA.

In summary, the flowchart for the MSHHOTSA algorithm is presented in Fig 7.

### 3.3 Pseudo-code of the MSHHOTSA algorithm

**Algorithm 1:** Multi-Strategy Hybrid Harris Hawks Tunicate Swarm Optimization Algorithm (MSHHOTSA)

**Input:** Population size: *N*, Search space dimension: *d*, Maximum number of iterations: *x*_max_

, lb, ub, *P*_min_, *P*_max_, *c*_1_,*c*_2_,*c*_3_,*r* = rand()

**Output:** $\vec{P_{P}(x*)}$

1: Initializing population $\vec{P_{P}(x)}$

2: Generate hyperbolic tangent domain function values according to Eq (9) and Eq (10)

3: Generate the nonlinear convergence factor function values according to Eq (12) and Eq (13)

4: Calculate the fitness of each tunicate individual and get the current optimal tunicate individual $\vec{P_{P}(x*)}$

5: **WHILE** (*x* < *x*_max_+1)

6: **FOR1** $i=1:\mathrm{size}(\vec{P_{P}(x)},1)$

7:  **FOR2** $j=1:\mathrm{size}(\vec{P_{P}(x)},2)$

8:  Using Eq (8) and Eq (16) to calculate the parameters $\vec{A}$ and *E*

9:  **IF1** (|*E*|≥1)

10:   **IF2** (*c*_3_≥0.5)

11:    Using Eq (6)-1 to calculate the position of the tunicate individual

12:   **ELSE**

13:    Using Eq (6)-2 to calculate the position of the tunicate individual

14:   **END IF2**

15:   Using Eq (14) to calculate the new tunicate individual position (TSA)

16:  **ELSEIF1** (|*E*|≥0.5 and *r*≥0.5)

17:   Using Eq (17) to calculate the new tunicate individual position (HHO)

18:  **ELSEIF1** (|*E*|≥0.5 and *r*<0.5)

19:   Using Eq (19) and Eq (20) to calculate the new tunic individual position (HHO)

20:  **ELSEIF1** (|*E*|<0.5 and *r*≥0.5)

21:   Using Eq (18) to calculate the new tunicate individual position (HHO)

22:  **ELSEIF1** (|*E*|<0.5 and *r*<0.5)

23:   Using Eq (21) to calculate the new tunicate individual position (HHO)

24:  **END IF1**

25: **END FOR2**

26:  Checking and fix the boundaries

27:  Calculate the fitness value of each tunicate individual

28:  Determine who is the better captive individual generated by TSA and HHO algorithms

29:  Updating the optimal tunicate

30:  **END FOR1**

31:  *x* = *x*+1

32: **END WHILE.**

### 3.4 Computational complexity

This subsection primarily analyzes the time complexity and space complexity of the proposed MSHHOTSA algorithm in this paper.

#### 3.4.1 Time complexity

Similar to other population-based metaheuristic algorithms, the MSHHOTSA algorithm’s population initialization has a time complexity of *O*(*N*×*d*). Including the hyperbolic tangent domain and the non-linear convergence factor in MSHHOTSA necessitates a separate calculation of their function values before each iteration. Consequently, the time complexity for computing the fitness function of each individual in the population becomes $O(N\times d\times x_{\max}+x_{\max}+x_{\max})$. Where *N* represents the population size, *d* represents the search space dimension, and *x*_max_ represents the maximum number of iterations.

In conclusion, the total time complexity of MSHHOTSA algorithm is $O(N\times d\times x_{\max}+x_{\max}+x_{\max})$.

#### 3.4.2 Space complexity

Throughout the algorithm’s iterative process, the population initialization step in MSHHOTSA consumes the most significant amount of space. As a result, the total space complexity of MSHHOTSA is *O*(*N*×*d*).

## 4. Experimental results and discussion

To assess the superior optimization performance of the proposed hybrid algorithm (MSHHOTSA) in solving complex functions, we conduct simulations and comparisons with seven other meta-heuristic algorithms (BOA, GWO, MVO, HHO, TSA, ASO, and WOA) using eight standard benchmark functions and the CEC2019 benchmark functions, while maintaining consistent basic parameters for each algorithm.

### 4.1 Experiments setup

**(1) Algorithm experimental environment**

Operating system: 64-bit Windows 11

CPU: 12th Gen Intel(R) Core(TM) i5-12500H 2.50 GHz Memory: 8G

**(2) Parameter settings**

In order to ensure the objective fairness of MSHHOTSA and other algorithms in comparative experiments, the basic parameters of each algorithm are set as shown in Table 1.

**Table 1:** Parameter settings.

Algorithm	Parameter	Value
All algorithms	Population size	50
	Maximum iterations	1000
	Number of experiments	30
ASO	Depth weight *α*	50
	Multiplier weight *β*	0.2
	Attractive upper limit *h*_max_	1.1
	Attractive lower limit *h*_min_	1.24
BOA	Probability switch *p*	0.8
	Power exponent parameter *a*	0.1
	Sensory modality *c*	0.01
MVO	WEP_max_	1
	WEP_min_	0.2
GWO	Convergence factor *a*	2→0
	*r*_1_, *r*_2_	Random
WOA	Convergence factor *a*	2→0
	*r*_1_, *r*_2_	Random
HHO	Convergence factor *E*_1_	2→0
	Initial state of its energy *E*_0_	[–1, 1]
	Levy factor *β*	1.5
TSA	The initial speed	1
	The subordinate speed	4
MSHHOTSA	Convergence factor *E*_1_	2→0
	Initial state of its energy *E*_0_	[–1, 1]
	The initial speed *P*_min_	1
	The subordinate speed *P*_max_	4
	*c*_1_, *c*_2_, *c*_3_	Random

**(3) Benchmark functions**

To demonstrate the superior performance of MSHHOTSA in solving complex functions, we conduct simulation experiments using 18 benchmark functions. The details of these benchmark functions can be found in Tables 2 and 3. Table 2 presents eight standard benchmark functions, while Table 3 lists the CEC2019 benchmark functions.

**Table 2:** Eight commonly used benchmark functions.

Index	Function name	Unimodal or Multimodal	lb	ub
*F*_1_	Bent Cigar	Unimodal	-100	100
*F*_*2*_	High Conditioned Elliptic	Unimodal	-100	100
*F*_3_	Stepint	Unimodal	-5.12	5.12
*F*_4_	Brown	Unimodal	-1	4
*F*_5_	Expanded Schaffer	Multimodal	-100	100
*F*_6_	Alpine N.1	Multimodal	-10	10
*F*_7_	Periodic	Multimodal	-10	10
*F*_8_	Trignometric 2	Multimodal	-500	500

**Table 3:** CEC2019 benchmark functions.

Index	Function name	*F*(x*)	Search space
cec01	Storn’s Chebyshev Polynomial Fitting Problem	1	[–8192, 8192]
cec02	Inverse Hilbert Matrix Problem	1	[–16384, 16384]
cec03	Lennard-Jones Minimum Energy Cluster	1	[–4, 4]
cec04	Rastrigin’s Function	1	[–100, 100]
cec05	Griewangk’s Function	1	[–100, 100]
cec06	Weierstrass Function	1	[–100, 100]
cec07	Modified Schwefel’s Function	1	[–100, 100]
cec08	Expanded Schaffer’s F6 Function	1	[–100, 100]
cec09	Happy Cat Function	1	[–100, 100]
cec10	Ackley Function	1	[–100, 100]

### 4.2 Result analysis of 8 benchmark functions

To evaluate the superior convergence and global search performance of MSHHOTSA, we selected four unimodal functions and four multimodal functions, subjecting them to the same conditions for analyzing the algorithm’s local exploitation and global exploration capabilities. The optimization results of the eight metaheuristics for the eight benchmark functions are presented in Table 4.

**Table 4:** The optimization results of each algorithm for the 8 benchmark functions.

Func.	Index	BOA	GWO	MVO	HHO	TSA	ASO	WOA	MSHHOTSA
*F*_1_	Mean	1.90E-14	4.33E-64	1.49E+05	2.63E-189	2.83E-46	1.79E+03	1.28E-165	**2.02E-203**
	Std	7.69E-16	1.29E-63	4.16E+04	0.00E+00	6.57E-46	2.18E+03	0.00E+00	**0.00E+00**
*F*_2_	Mean	1.80E-14	1.05E-67	5.02E+06	2.30E-189	1.14E-48	7.50E+03	7.23E-168	**1.47E-205**
	Std	1.07E-15	1.71E-67	1.63E+06	0.00E+00	2.66E-48	4.51E+03	0.00E+00	**0.00E+00**
*F*_3_	Mean	-3.23E+01	-1.40E+02	-1.45E+02	-1.55E+02	-8.06E+01	**-1.19E+02**	-1.55E+02	-1.55E+02
	Std	6.69E+00	6.38E+00	3.49E+00	0.00E+00	1.04E+01	3.33E+00	0.00E+00	**0.00E+00**
*F*_4_	Mean	1.44E-14	9.34E-73	4.46E-04	8.89E-196	2.10E-54	4.65E-21	3.38E-175	**1.97E-218**
	Std	1.30E-15	3.89E-72	1.15E-04	0.00E+00	8.81E-54	6.64E-21	0.00E+00	**0.00E+00**
*F*_5_	Mean	1.06E+01	5.08E+00	1.12E+01	0.00E+00	1.18E+01	9.82E+00	1.47E+00	**0.00E+00**
	Std	5.40E-01	1.72E+00	7.19E-01	0.00E+00	6.98E-01	6.68E-01	2.95E+00	**0.00E+00**
*F*_6_	Mean	1.24E-14	6.06E-06	3.15E+00	1.31E-06	2.49E+01	5.79E-11	9.11E-111	**3.58E-112**
	Std	2.16E-14	1.26E-05	1.71E+00	6.90E-06	5.81E+00	5.50E-11	3.92E-110	**1.24E-111**
*F*_7_	Mean	7.53E+00	1.39E+00	1.00E+00	9.00E-01	3.60E+00	1.00E+00	1.00E+00	**9.00E-01**
	Std	6.64E-01	1.01E+00	4.54E-04	4.52E-16	7.86E-01	5.83E-17	1.36E-01	**4.52E-16**
*F*_8_	Mean	8.10E+01	2.24E+01	1.71E+02	**1.00E+00**	1.02E+02	1.35E+01	5.07E+01	3.40E+01
	Std	7.78E+00	4.29E+00	3.09E+01	**1.31E-04**	2.36E+01	7.42E+00	1.85E+01	1.65E+01

From Table 4, it is evident that under the same constraints:

1. Considering the overall optimization results of the algorithm on eight benchmark functions, MSHHOTSA exhibits a more minor mean and standard deviation in most cases. This indicates that the improved algorithm demonstrates better convergence and optimization performance than other algorithms.
2. Judging from the optimization results of the algorithm for unimodal functions (*F*_1_~*F*_4_): Compared to the other seven metaheuristic algorithms, MSHHOTSA achieves the best performance metrics (most minor mean and standard deviation) on three unimodal benchmark functions (*F*_1_, *F*_2_, and *F*_4_). Additionally, for the mean metric on *F*_3_, MSHHOTSA ranks fourth. However, there is no significant difference compared to the top-ranking algorithm, and MSHHOTSA exhibits better stability with a minor standard deviation. This indicates that MSHHOTSA has superior performance and stability. The experimental results of MSHHOTSA on the unimodal functions indicate its superior performance in local development.
3. Judging from the optimization results of the algorithm for multimodal functions (*F*_5_~*F*_8_): It is evident that except for *F*_8_, where the optimization results of MSHHOTSA are not the best. The outcomes of the three functions (*F*_5_~*F*_7_) are significantly superior to those achieved by the other seven comparison algorithms. While the optimization result of the improved algorithm on *F*_8_ is only ranked 4th, an analysis of the optimization results of TSA, HHO, and MSAHHOTSA algorithms reveals that MSHHOTSA falls between HHO and TSA. This suggests that the performance adjustment using HHO optimization is effective for TSA. Consequently, considering the overall performance indicators of the four multimodal functions, MSHHOTSA demonstrates higher global exploration capabilities than the comparison algorithms.

**Fig 8:**
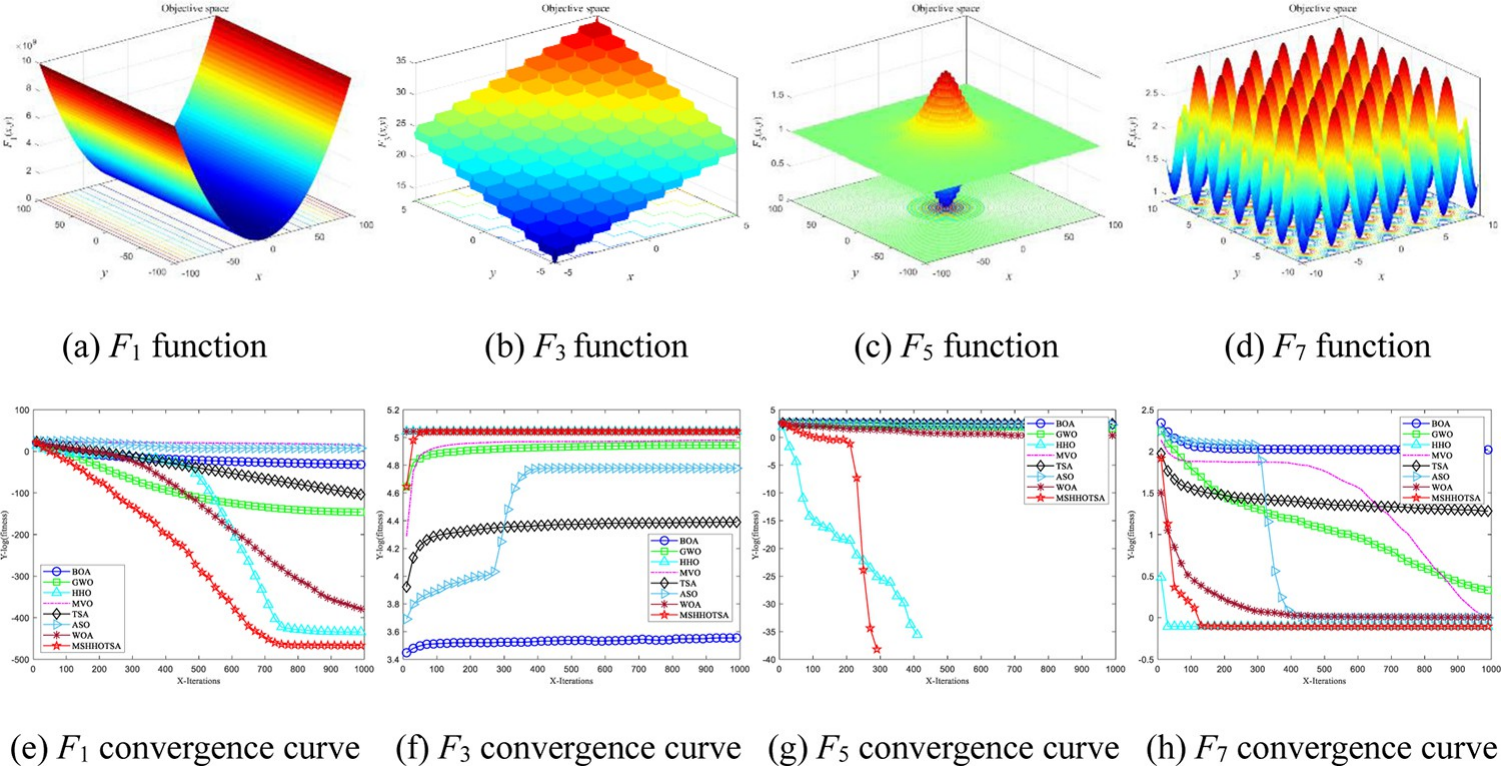
Iterative optimization convergence curve of each algorithm.

To vividly compare and analyze the superiority of MSHHOTSA over the seven meta-heuristic algorithms, we selected two unimodal and two multimodal functions to plot the optimal convergence curves of each algorithm under 1000 iterations, as depicted in Fig 8. The results from Fig 8 indicate that MSHHOTSA exhibits a faster convergence speed and higher convergence accuracy compared to the five comparison algorithms in the optimization iteration process for both unimodal and multimodal functions. This optimization iteration curve verifies the improved algorithm’s superior optimization performance in solving complex functions.

### 4.3 Result analysis of CEC2019 benchmark functions

To further validate MSHHOTSA’s superior generalization performance in tackling complex functions, we conducted a comparative analysis of the optimized results using the CEC2019 benchmark functions with the same constraints. Table 5 shows the optimization results of each algorithm on CEC2019 benchmark functions.

**Table 5:** The optimization results of each algorithm for the CEC2019 benchmark functions.

Function	Index	BOA	GWO	MVO	HHO	TSA	ASO	WOA	MSHHOTSA
cec01	Mean	1.00E+00	1.58E+01	4.59E+01	1.00E+00	1.06E+02	1.76E+02	3.99E+03	**1.00E+00**
	Rank	3	5	8	2	4	6	7	1
	Std	0.00E+00	2.59E+01	8.96E+01	**0.00E+00**	1.87E+02	1.77E+02	5.62E+03	2.48E-14
cec02	Mean	5.00E+00	5.47E+00	5.68E+00	4.97E+00	7.64E+00	**3.11E+00**	1.06E+01	5.00E+00
	Rank	4	5	6	2	7	1	8	3
	Std	2.91E-03	9.48E-01	9.00E-01	8.04E-02	1.86E+00	3.26E-01	7.54E+00	**0.00E+00**
cec03	Mean	6.34E+00	1.23E+01	1.24E+01	4.49E+00	1.27E+01	1.26E+01	1.19E+01	**4.32E+00**
	Rank	3	5	6	2	8	7	4	1
	Std	1.03E+00	8.09E-01	5.96E-01	1.98E+00	**7.61E-04**	3.65E-01	9.95E-01	2.24E+00
cec04	Mean	7.24E+01	**1.39E+01**	1.81E+01	4.65E+01	4.80E+01	2.01E+01	5.42E+01	2.94E+01
	Rank	8	1	2	5	6	3	7	4
	Std	1.09E+01	**5.50E+00**	7.23E+00	1.37E+01	1.49E+01	9.23E+00	1.72E+01	1.89E+01
cec05	Mean	1.02E+02	1.69E+00	1.31E+00	1.95E+00	2.52E+01	**1.00E+00**	1.97E+00	1.36E+00
	Rank	2	5	3	6	8	1	7	4
	Std	3.13E+01	5.67E-01	1.26E-01	2.62E-01	2.04E+01	**4.10E-03**	3.70E-01	2.80E+00
cec06	Mean	7.74E+00	2.50E+00	3.33E+00	7.39E+00	7.57E+00	**1.55E+00**	8.74E+00	5.28E+00
	Rank	7	2	3	5	6	1	8	4
	Std	**7.73E-01**	1.02E+00	1.73E+00	1.76E+00	1.99E+00	9.05E-01	1.72E+00	1.55E+00
cec07	Mean	1.76E+03	**6.42E+02**	6.85E+02	1.17E+03	1.17E+03	8.91E+02	1.25E+03	8.92E+02
	Rank	8	1	2	6	5	3	7	4
	Std	**1.37E+02**	4.13E+02	2.36E+02	2.37E+02	3.11E+02	2.51E+02	3.70E+02	2.80E+02
cec08	Mean	4.64E+00	**3.48E+00**	3.64E+00	4.60E+00	4.49E+00	3.90E+00	4.52E+00	4.04E+00
	Rank	8	1	2	7	5	3	6	4
	Std	**2.21E-01**	4.78E-01	5.89E-01	2.84E-01	4.14E-01	5.63E-01	3.54E-01	3.08E-01
cec09	Mean	3.88E+00	1.16E+00	1.21E+00	1.38E+00	1.77E+00	1.12E+00	1.40E+00	**1.02E+00**
	Rank	8	3	4	5	7	2	6	1
	Std	7.13E-01	7.06E-02	**6.00E-02**	1.56E-01	8.49E-01	7.14E-02	2.53E-01	1.50E-01
cec10	Mean	2.13E+01	2.10E+01	2.10E+01	2.11E+01	2.14E+01	2.10E+01	2.11E+01	**2.10E+01**
	Rank	7	4	3	6	8	2	5	1
	Std	5.64E-01	1.96E+00	4.05E-02	6.43E-02	9.01E-02	**1.43E-03**	1.07E-01	1.72E-01
Total	Rank	5.90	3.30	4.00	4.70	6.50	3.00	5.80	**2.80**

From Table 5, under the same experimental conditions:

For MSHHOTSA, as a hybrid algorithm, its performance in terms of mean and standard deviation indicators is significantly superior to that of the HHO and TSA algorithms. This suggests that the hybrid algorithm effectively combines the advantages of HHO and TSA algorithms, leading to an overall performance enhancement.

Regarding the mean value indicators, MSHHOTSA demonstrates optimal performance on cec01, cec03, cec09, and cec10, ranking 3rd on cec02 and 4th on the remaining five benchmark functions. Similarly, concerning standard deviation indicators, the ranking of the MSHHOTSA algorithm on CEC2019 benchmark functions could be further improved. This indicates that no single algorithm can address all optimization problems. Therefore, when dealing with different optimization problems, one should consider the specific requirements and conduct comparative experiments to select a more suitable algorithm for solving them.

In conclusion, although the performance of the MSHHOTSA algorithm may require enhancement on certain benchmark functions, considering the overall ranking based on average value indicators, the MSHHOTSA algorithm still exhibits superior performance. This scalability implies that the algorithm has broader potential for application and can achieve excellent results in handling various optimization problems.

**Fig 9:**
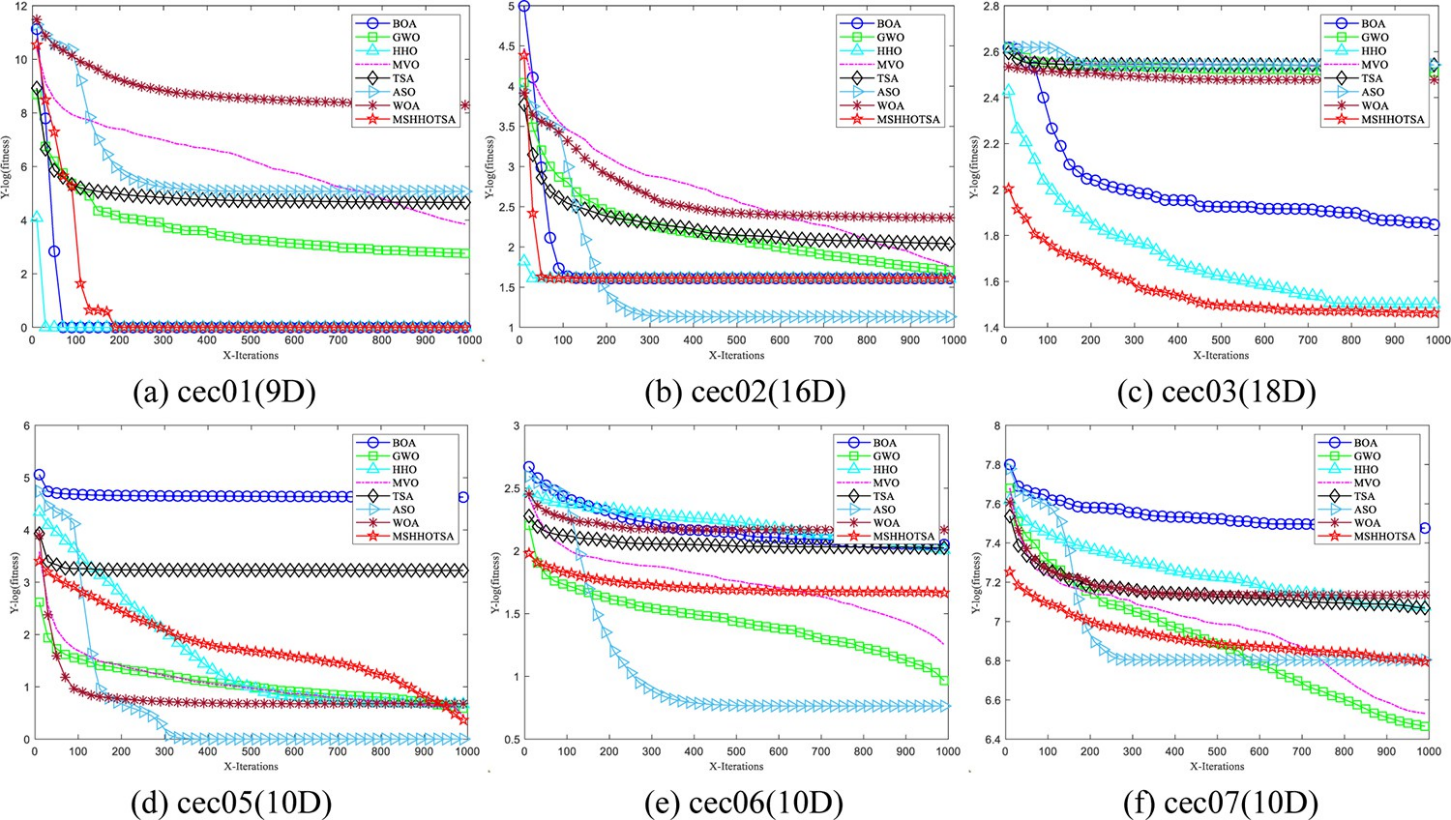
Iterative convergence curves of each algorithm for the CEC2019 benchmark functions.

Fig 9 shows the convergence curves of eight algorithms on CEC2019 benchmark functions aimed at analyzing and exploring the convergence performance of the MSHHOTSA algorithm and other algorithms.

From Fig 9, under the same experimental conditions:

The MSHHOTSA algorithm achieved the best convergence accuracy on cec01, cec03, cec09, and cec10. The ASO algorithm performed best on cec02, cec05, and cec06. The GWO algorithm performed best on cec07 and cec08.

However, despite achieving the best convergence accuracy on specific functions, the MSHHOTSA algorithm does not exhibit the fastest overall convergence speed. This can be attributed to two main factors: (1) the MSHHOTSA algorithm is a hybrid of HHO and TSA algorithms, and it tends to emphasize global exploration in the early stages of the iteration, leading to slower algorithm convergence speed. (2) The complexity analysis in Subsection 3.4.1 reveals that the MSHHOTSA algorithm has a relatively higher time complexity than specific individual metaheuristic algorithms, leading to a slower convergence speed.

From Fig 9, it can also be observed that the performance of the MSHHOTSA algorithm shows minimal changes after 100 iterations. This phenomenon can be attributed to the following reasons: (1) The algorithm has already converged to the global optimum, leading to stable performance, as evident in the case of the cec01 function. (2) The global exploration capability of the algorithm is limited, causing it to get trapped in local optima. As a result, no larger step sizes facilitate escaping from the current local optima, as observed in cec02, cec06, and cec08. (3) The inherent complexity of the problem requires a more significant number of iterations for extensive global search in the solution space and gradual approximation towards the global optimum, as seen in cec03, cec05, cec09, and cec10.

In addition, Fig 9 illustrates that the optimization performance of the MSHHOTSA algorithm before the 100th iteration is suboptimal. This can be attributed to the following reasons:

1. The initial population is not well-distributed. The effectiveness of any optimization algorithm, including MSHHOTSA, to a large extent, depends on the initial distribution of the population. When the initial population of MSHHOTSA is not well-distributed, the algorithm may become trapped in local optima and struggle to escape, thereby limiting its global search capability during the early iterations. Consequently, suboptimal performance is observed before the 100th iteration.
2. The hybrid algorithm emphasizes more on global exploration in the early stages. MSHHOTSA combines different search strategies, so it tends to explore globally during the initial iterations to search for more potential solutions in the search space. However, this extensive global exploration hinders the algorithm from rapidly converging to the optimal solution.
3. The hyperbolic tangent domain restricts the step size change of the algorithm, and the nonlinear fast convergence factor drives the algorithm to converge quickly. The hyperbolic tangent domain is used to correct the individual tunicate position and imposes a rule restriction on the step size change of the algorithm. This restriction causes the MSHHOTSA algorithm to move slowly in the search space. At the same time, the nonlinear fast convergence factor decreases rapidly, and the driving algorithm tends to develop locally, eventually leading to poor optimization performance of the MSHHOTSA algorithm in the early stage.

In the later stages of iteration, the nonlinear fast convergence factor shows the nonlinear increase and decrease fluctuations. Meanwhile, the hybrid algorithm and hyperbolic tangent domain continuously update new populations, improving the diversity of the population. Therefore, through cooperation and competition among the three improvement strategies, the MSHHOTSA algorithm can adjust its behavior at different stages, thus enhancing its overall optimization performance.

### 4.4 Result analysis of the algorithm running time

In assessing the algorithm’s performance, it demonstrates improved accuracy and faster convergence in the curve. These outcomes signify superior optimization performance within numerical theory when addressing the problem. However, it is essential to note that as any algorithm’s complexity increases, its optimization performance may also be somewhat enhanced. Therefore, it is significant to investigate the algorithm’s specific running time when tackling complex problems to evaluate its practical applicability effectively.

**Fig 10:**
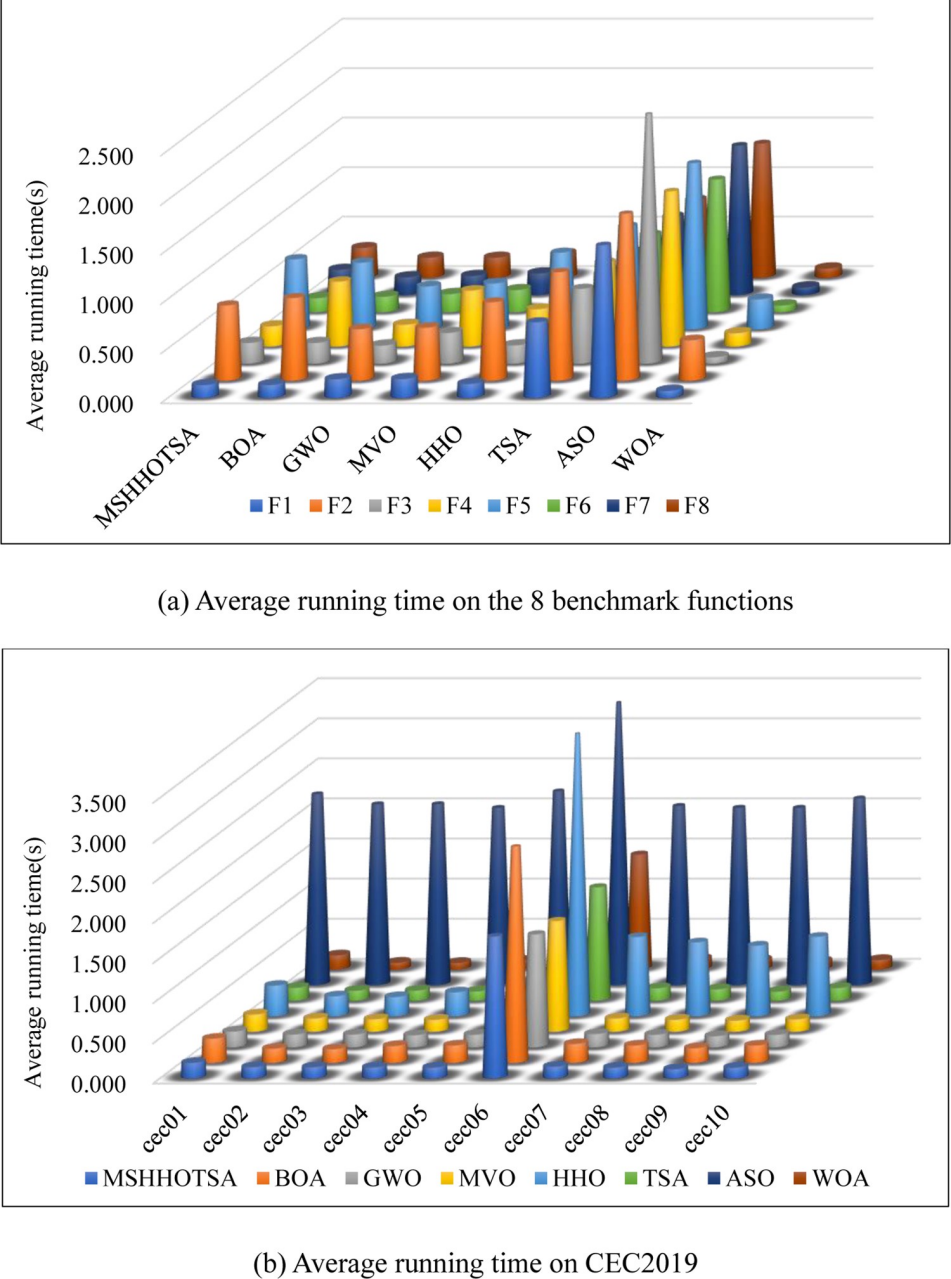
Comparison of average running time of each algorithm.

To verify whether MSHHOTSA exhibits a faster runtime and better optimization performance, the runtimes of each algorithm were recorded while solving 18 benchmark functions in 30 experiments. The corresponding average runtimes of the algorithms are illustrated in Fig 10. Specifically, Fig 10(A) represents the average running time of each algorithm on the eight benchmark functions. At the same time, Fig 10(B) displays the average running time of each algorithm on the CEC2019 benchmark functions.

Fig 10(A) shows that when solving the *F*_1_ benchmark function, all eight metaheuristic algorithms exhibit the shortest runtime when solving other benchmark functions. Additionally, the ASO algorithm performs the worst among these eight algorithms, while the WOA algorithm demonstrates the best performance. Notably, our proposed MSHHOTSA algorithm ranks third in runtime among the eight algorithms. Similarly, when considering the CEC2019 benchmark functions, the ASO algorithm still performs poorly, and the WOA algorithm remains the best. Our proposed MSHHOTSA algorithm ranks fifth in runtime among the eight algorithms.

From the above results, it can be observed that the MSHHOTSA algorithm does not show significant differences in runtime when solving the 18 benchmark functions. This can be attributed to the integration of the three improvement strategies. Despite improving the algorithm’s accuracy, these strategies contribute to its increased computational complexity, resulting in longer runtime. Nevertheless, the superior performance of MSHHOTSA over other algorithms on certain benchmark functions may be influenced by the computer’s operating conditions and random numbers. It is worth noting that, compared to the ASO algorithm, the MSHHOTSA algorithm exhibits faster runtime.

### 4.5 Result analysis of the Wilcoxon’s rank sum test

The mean and standard deviation obtained by solving the benchmark function through 30 independent experiments can confirm the improved algorithm’s overall better optimization performance. However, it cannot determine the significance of the running results for each algorithm. To comprehensively showcase the superior performance of MSHHOTSA, Wilcoxon’s rank sum test is conducted at a 5% significance level from a statistical perspective [69, 70], and the *p*-value is calculated. The test results are presented in Table 6, where MSHHOTSA is compared with other algorithms. The symbols “+”, “-”, and “=” indicate that the improved algorithm performs better than, worse than, or equivalently to the comparison algorithm, respectively. “N/A” denotes “not applicable”, signifying no significant performance difference is observed between the two algorithms.

**Table 6:** Wilcoxon’s rank sum test results.

Function	MSHHOTSA vs. BOA	MSHHOTSA vs. GWO	MSHHOTSA vs. MVO	MSHHOTSA vs. HHO	MSHHOTSA vs. TSA	MSHHOTSA vs. ASO	MSHHOTSA vs. WOA
*F*_1_	6.50E-218	9.47E-100	7.51E-314	9.20E-46	3.64E-178	8.42E-300	1.39E-56
*F*_*2*_	4.42E-231	9.68E-91	1.22E-314	2.88E-43	1.16E-171	5.97E-303	4.32E-50
*F*_3_	N/A	N/A	N/A	2.72E-17	N/A	N/A	9.73E-17
*F*_4_	1.42E-266	8.08E-98	2.87E-316	3.62E-49	5.57E-183	2.02E-287	2.13E-53
*F*_5_	N/A	1.72E-306	N/A	1.75E-02	N/A	1.45E-317	8.10E-264
*F*_6_	4.57E-253	2.41E-263	6.58E-321	6.03E-256	1.30E-322	3.49E-284	7.19E-39
*F*_7_	N/A	1.79E-303	3.97E-308	7.04E-03	2.13E-312	1.17E-246	1.82E-219
*F*_8_	1.43E-280	2.45E-07	6.56E-315	2.99E-303	1.22E-294	1.76E-23	2.46E-186
cec01	6.45E-204	5.85E-210	3.34E-245	1.28E-79	1.40E-212	9.55E-232	2.15E-295
cec02	4.62E-160	5.58E-292	5.57E-300	1.84E-136	3.62E-292	2.90E-142	1.01E-300
cec03	1.79E-297	N/A	N/A	9.88E-85	N/A	N/A	N/A
cec04	N/A	7.82E-95	5.51E-21	1.75E-294	2.70E-258	1.75E-148	2.89E-294
cec05	N/A	1.66E-128	1.83E-116	7.04E-21	8.24E-297	5.09E-176	1.86E-199
cec06	N/A	1.62E-200	4.40E-08	N/A	N/A	3.58E-164	N/A
cec07	N/A	4.85E-04	3.29E-15	1.85E-283	1.61E-249	1.80E-85	2.92E-270
cec08	N/A	2.44E-104	7.02E-42	N/A	2.39E-321	3.81E-87	1.31E-320
cec09	6.90E-323	4.98E-14	**5.78E-02**	2.09E-10	4.65E-71	1.33E-37	4.72E-04
cec10	2.33E-210	4.45E-05	4.82E-207	2.10E-38	1.31E-268	2.00E-52	9.42E-40
+/ = /-	10/8/0	16/2/0	14/3/1	16/2/0	14/4/0	16/2/0	16/2/0

Table 6 shows that MSHHOTSA has more “+” occurrences than other metaheuristic algorithms, indicating statistically significant differences between MSHHOTSA and all competing algorithms. Furthermore, MSHHOTSA performs better on benchmark functions marked with “+”. Conversely, there are more “=” when comparing MSHHOTSA with the BOA algorithm, suggesting no statistically significant difference between the two algorithms on benchmark functions marked with “=“. In particular, on the cec09 benchmark function, MSHHOTSA exhibits “-” when compared to the MVO algorithm, indicating inferior performance on cec09 compared to MVO. However, considering the overall performance, MSHHOTSA consistently displays better results. Hence, from a statistical standpoint, the MSHHOTSA algorithm demonstrates better statistical significance than other algorithms, highlighting its superior optimization convergence and robustness.

### 4.6 Discussion

Based on the experimental results from Sections 4.2 to 4.5, we can conclude that, overall, the MSHHOTSA algorithm demonstrates better optimization performance in solving eight standard benchmark functions and CEC2019 benchmark functions. This is reflected in smaller average values, standard deviations, and higher overall ranking indicators. It indicates that the MSHHOTSA algorithm performs well in solving multiple optimization problems, and its advantages are consistently demonstrated. Furthermore, smaller Wilcoxon rank sum test p-values also validate the superior statistical performance of MSHHOTSA.

However, it is worth noting that when we analyze the performance of the MSHHOTSA algorithm separately for each benchmark function, we find that the algorithm does not consistently achieve the best performance on every benchmark function. Below, we will provide a detailed analysis of the main reasons leading to the suboptimal performance of the MSHHOTSA algorithm on certain benchmark functions. In future research, readers can use these shortcomings to improve the algorithm’s performance.

1. Adding the hyperbolic tangent domain and the nonlinear fast convergence factor has increased algorithm complexity, decreasing the algorithm’s convergence speed. While these three new improvement strategies enable the MSHHOTSA algorithm to achieve better convergence accuracy on certain benchmark functions, they also come at the cost of increased algorithm complexity, leading to a decrease in the convergence speed of MSHHOTSA.
2. The two TSA and HHO algorithms are mixed, making the hybrid algorithm more inclined to global exploration. The MSHHOTSA algorithm introduces the population update mechanism of the HHO algorithm into the TSA algorithm, which increases the diversity of the new population and promotes the algorithm to explore further solutions, but also limits the local mining performance of MSHHOTSA.
3. The nonlinear fast convergence factor prevents the algorithm from quickly converging towards local exploitation in the later iteration stages. As observed from Fig 6, our proposed nonlinear fast convergence factor exhibits fluctuations in the middle and later stages of algorithm iteration. This behavior causes the algorithm to continue jumping around the global optimal solution even after finding it, leading to a suboptimal convergence speed of MSHHOTSA in the later stages.
4. The combined effect of the hyperbolic tangent domain and the nonlinear fast-convergence factor makes it challenging for MSHHOTSA to escape local optima in the early stages of iteration. Introducing the hyperbolic tangent domain restricts the step size change of the MSHHOTSA algorithm, leading to slow movement in the search space. Simultaneously, the rapid decrease of our proposed nonlinear fast-convergence factor drives the algorithm towards local exploitation, ultimately causing MSHHOTSA to struggle in escaping local optima in the early stages of iteration.

Although the proposed MSHHOTSA algorithm still has many deficiencies, the coin has two sides, and scientific research is no exception. For the MSHHOTSA algorithm, if we unthinkingly pursue the excellent performance of the experimental results, we can also choose multiple sets of benchmark functions for experiments and finally select functions with good optimization results and put them in the paper’s experimental results. However, it is unfair and unscientific to select functions with good optimization results unthinkingly and put them in the experimental results. This also enlightens us that when researching algorithms, we should not only focus on the shortcomings of the algorithm but also discover the shining points of the algorithm. Whether it is the advantages or disadvantages of the algorithm, as long as we conduct a thorough analysis and discussion, we can slowly eliminate its shortcomings in the following research and promote the spiral performance of the algorithm we propose.

In conclusion, although the MSHHOTSA algorithm still has certain limitations when solving specific problems, it successfully addresses certain targeted issues. This indicates that our proposed three improvement strategies possess unique merits in enhancing algorithm performance. Thus, when refining other metaheuristic algorithms, these strategies can serve as valuable references for improvement.

Furthermore, when solving real-world optimization problems, conducting experiments with multiple comparative algorithms is essential. Doing so allows us to select the most suitable parameters and identify the algorithm that yields the best results for the given problem. This comprehensive approach will contribute to achieving optimal solutions in practical optimization scenarios.

## 5. MSHHOTSA for engineering optimization problems

Engineering optimization problems are objective optimization models commonly used in scientific and engineering applications to address real-world engineering challenges. Due to their nonlinear and strongly constrained characteristics, finding effective solutions becomes crucial in evaluating algorithm performance in practical scenarios. To assess the feasibility and applicability of the MSHHOTSA algorithm in solving engineering optimization problems, we consider three optimization problems [3, 8, 71, 72]: the tension/compression spring design problem, the pressure vessel design problem, the gear train design problem, the speed reducer design problem, and the parameters optimization of the proportional-integral-derivative (PID) controller. The algorithm’s performance is thoroughly investigated in these cases. Each algorithm is tested with a population size of 30, a maximum of 1000 iterations, and each set of experiments is conducted independently 30 times.

### 5.1 Tension/Compression spring design problem

**Fig 11:**
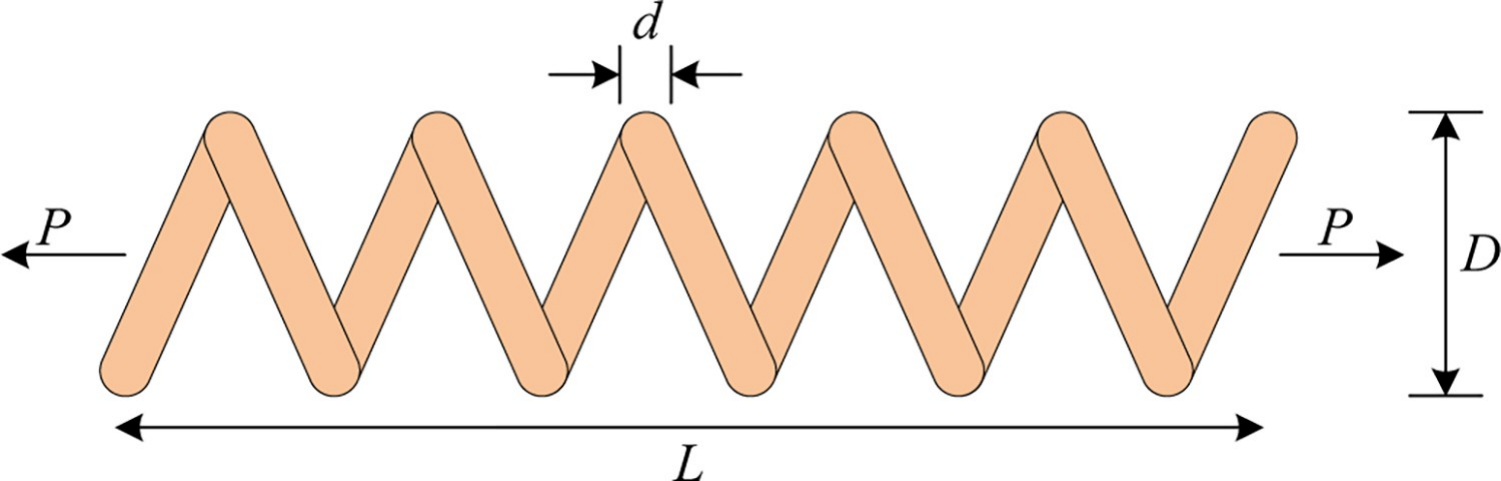
Tension/compression spring design problem.

The optimization objective of the extension/compression spring design problem is to minimize the spring mass [8, 71]. The topology of the problem is shown in Fig 11. In this problem, the depreciation of the spring mass, i.e., the optimization objective of the problem, is constrained by the minimum deviation, shear stress, impact frequency, outer diameter limit, and three decision variables: the average coil diameter *D*, the wire diameter *d*, and the number of active coils *P*.

Let $\vec{x}=[x_{1},x_{2},x_{3}]=[d,D,P]$, $f(\vec{x})$ is the mass of the spring, then the mathematical model of the extension/compression spring design problem can be described as follows.

$$\min\;f(\vec{x})=(x_{3}+2)x_{2}x_{1}^{2}$$ (22)

$$s.t.\{\begin{array}{l} g_{1}(\vec{x})=1-\frac{x_{2}^{3}x_{3}}{71785x_{1}^{4}}\leq 0\\ g_{2}(\vec{x})=\frac{4x_{2}^{2}-x_{1}x_{2}}{12566(x_{2}x_{1}^{3}-x_{1}^{4})}+\frac{1}{5108x_{1}^{2}}-1\leq 0\\ g_{3}(\vec{x})=1-\frac{140.45x_{1}}{x_{2}^{2}x_{3}}\leq 0\\ g_{4}(\vec{x})=\frac{x_{1}+x_{2}}{1.5}-1\leq 0 \end{array}$$ (23)

Where *x*_1_∈[0.05,2], *x*_2_∈[0.25,1.3], *x*_3_∈[2,15].

**Fig 12:**
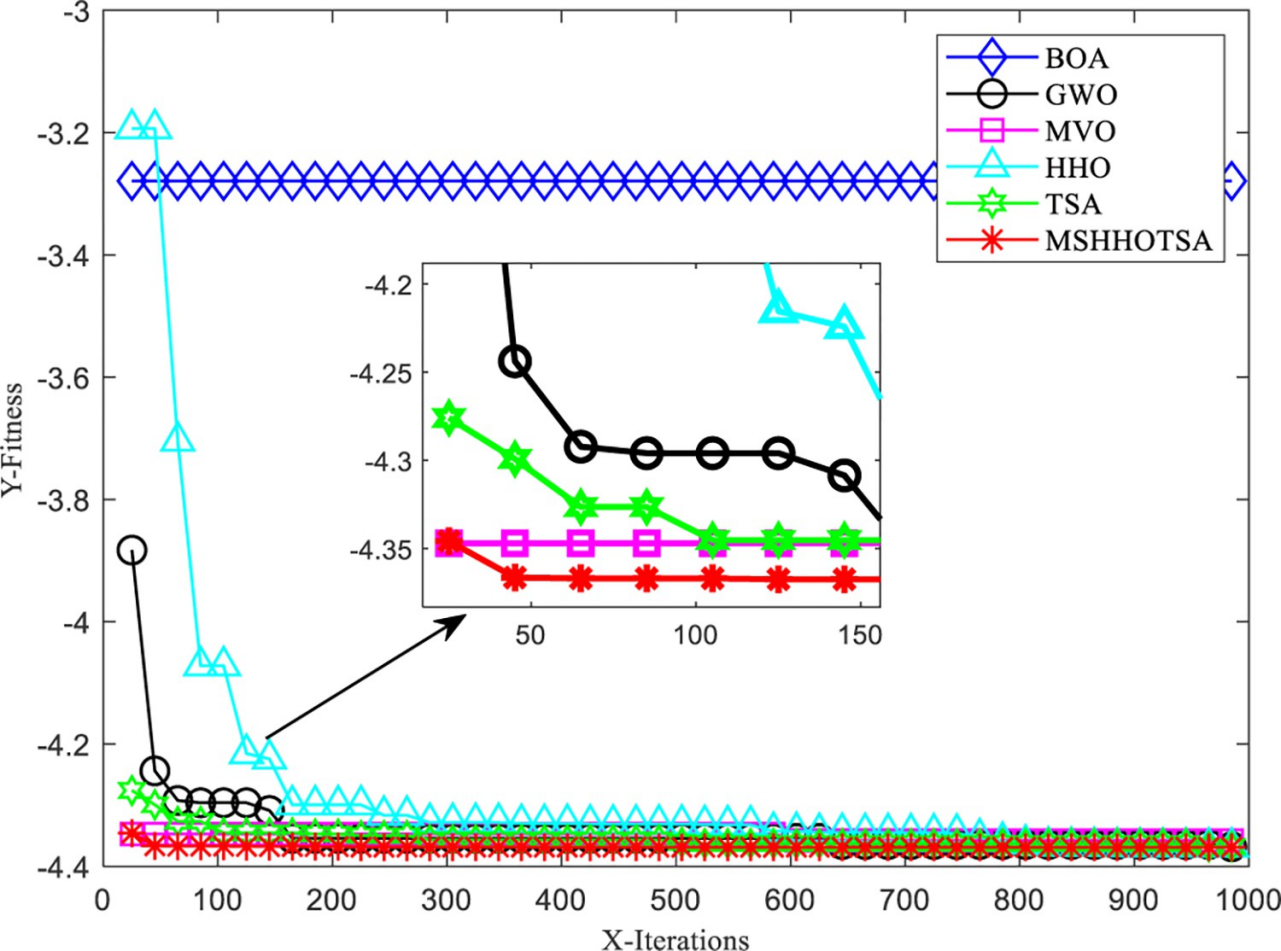
Convergence curves for each algorithm to solve the tension/compression spring design problem.

Table 7 and Fig 12 show the best-finding results of the six meta-heuristic algorithms for the extension/compression spring design problem under 30 independent experiments and the convergence curves of the finding for each algorithm in solving the problem, respectively.

**Table 7:** Optimal results of the different algorithms on the tension/compression spring design problem.

Algorithm	*d*	*D*	*P*	$f(\vec{x})$
BOA	0.076	0.858	5.675	0.037658
GWO	0.052	0.360	11.101	0.012669
MVO	0.050	0.316	14.165	0.012786
HHO	0.052	0.367	10.689	0.012670
TSA	0.052	0.366	10.790	0.012680
MSHHOTSA	0.052	0.354	11.476	**0.012666**

From Table 7, the optimal solution of MSHHOTSA for the same constraints is $\left[x_{1},x_{2},x_{3}]=[0.052,0.354,11.476\right]$ with the optimal value of $f(\vec{x})=0.012666$, which shows that MSHHOTSA is the best solution for reducing the mass of the extension/compression spring compared to the other compared algorithms. It is also clear from the analysis in Fig 12 that MSHHOTSA has a better optimization performance and a faster convergence rate when solving the tension/compression spring design problem.

### 5.2 Pressure vessel design problem

**Fig 13:**
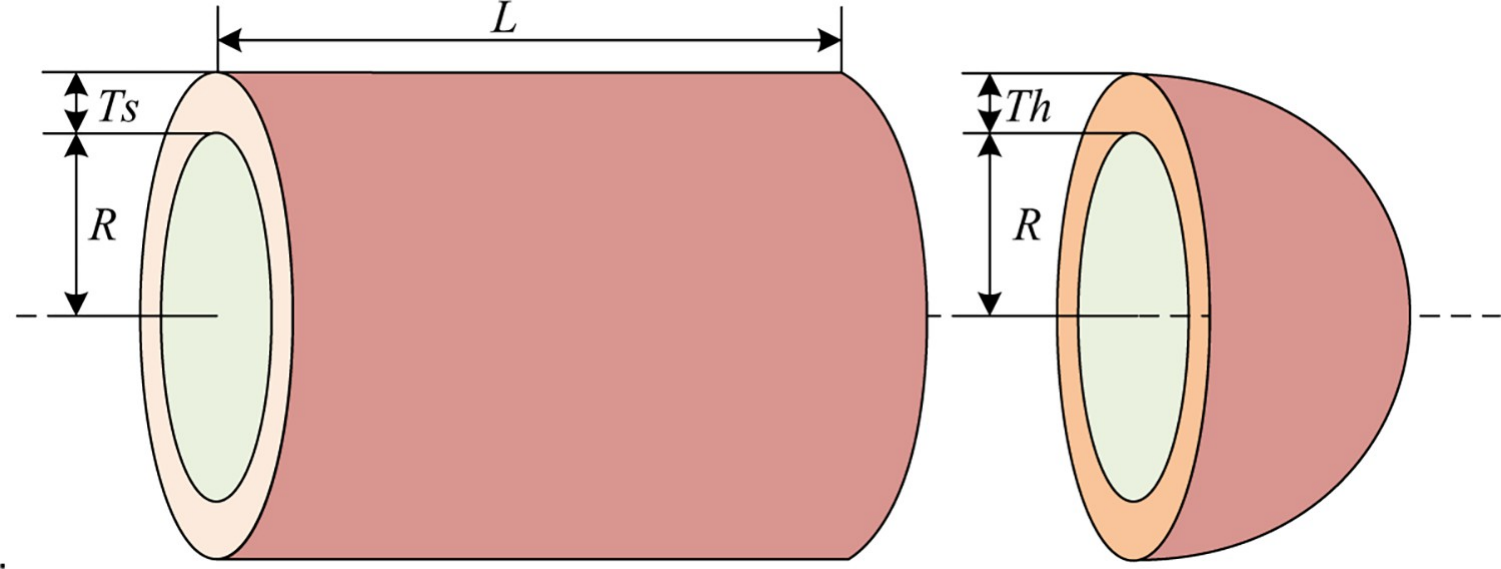
Pressure vessel design problem.

The optimization objective of the pressure vessel design problem is to minimize the total cost of fabricating the pressure vessel (materials, shaping, and welding) [8, 71]. The structural topology of the problem is shown in Fig 13. In this problem, the minimization of the fabrication cost, i.e., the optimization objective of the problem, is jointly influenced by four decision variables: the length of the interface of the cylindrical section *L*, the radius of entry *R*, the thickness of the shell *Ts*, and the thickness of the head *Th*.

Let $\vec{x}=[x_{1},x_{2},x_{3},x_{4}]=[T_{s},T_{h},R,L]$, $f(\vec{x})$ is the total cost of making the pressure vessel, then the mathematical model of the pressure vessel design problem is described as:

$$\min\;f(\vec{x})=0.6224x_{1}x_{3}x_{4}+1.7781x_{2}x_{3}^{2}+3.1661x_{1}^{2}x_{4}+19.84x_{1}^{2}x_{3}$$ (24)

$$s.t.\{\begin{array}{l} g_{1}(\vec{x})=-x_{1}+0.0193x_{3}\leq 0\\ g_{2}(\vec{x})=-x_{3}+0.00954x_{3}\leq 0\\ g_{3}(\vec{x})=-\pi x_{3}^{2}x_{4}-\frac{4}{3}\pi x_{3}^{3}+1296000\leq 0\\ g_{4}(\vec{x})=x_{4}-240\leq 0 \end{array}$$ (25)

Where *x*_1_∈[0,99], *x*_2_∈[0,99], *x*_3_∈[10,200], *x*_4_∈[10,200].

**Fig 14:**
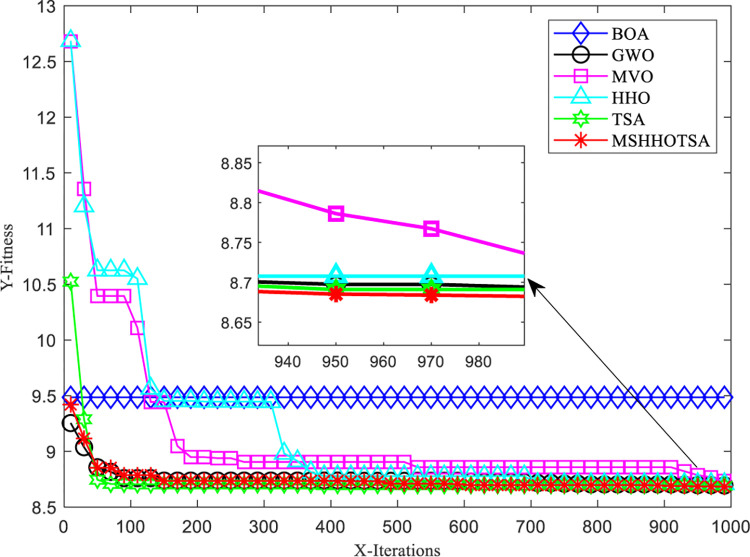
Convergence curves for each algorithm to solve the pressure vessel design problem.

Table 8 and Fig 14 show the best-finding results of the six metaheuristic algorithms for the pressure vessel design problem under 30 independent experiments and the convergence curves of the finding for each algorithm in solving the problem, respectively.

**Table 8:** Optimal results of the different algorithms on the pressure vessel design problem.

Algorithm	*T*_*s*_	*T*_*h*_	*R*	*L*	$f(\vec{x})$
BOA	1.13	1.59	57.07	57.07	13167.6336
GWO	0.82	0.40	42.28	174.47	5956.1007
MVO	0.85	0.42	43.99	155.49	6057.8781
HHO	0.83	0.41	42.28	174.42	6049.4608
TSA	0.78	0.39	40.35	200.00	5913.9777
MSHHOTSA	0.78	0.39	40.32	200.00	**5889.7831**

As shown in Table 8, under the same conditions, the MSHHOTSA achieves a smaller pressure vessel design production cost than the other five compared algorithms; also, the convergence curve in Fig 14 shows that the improved algorithm has a faster convergence speed and the ability to escape local extremes in solving pressure vessel design problems, which again shows the superior performance of MSHHOTSA in solving engineering problems. This again shows the superior performance of MSHHOTSA in solving engineering problems.

### 5.3 Gear train design problem

**Fig 15:**
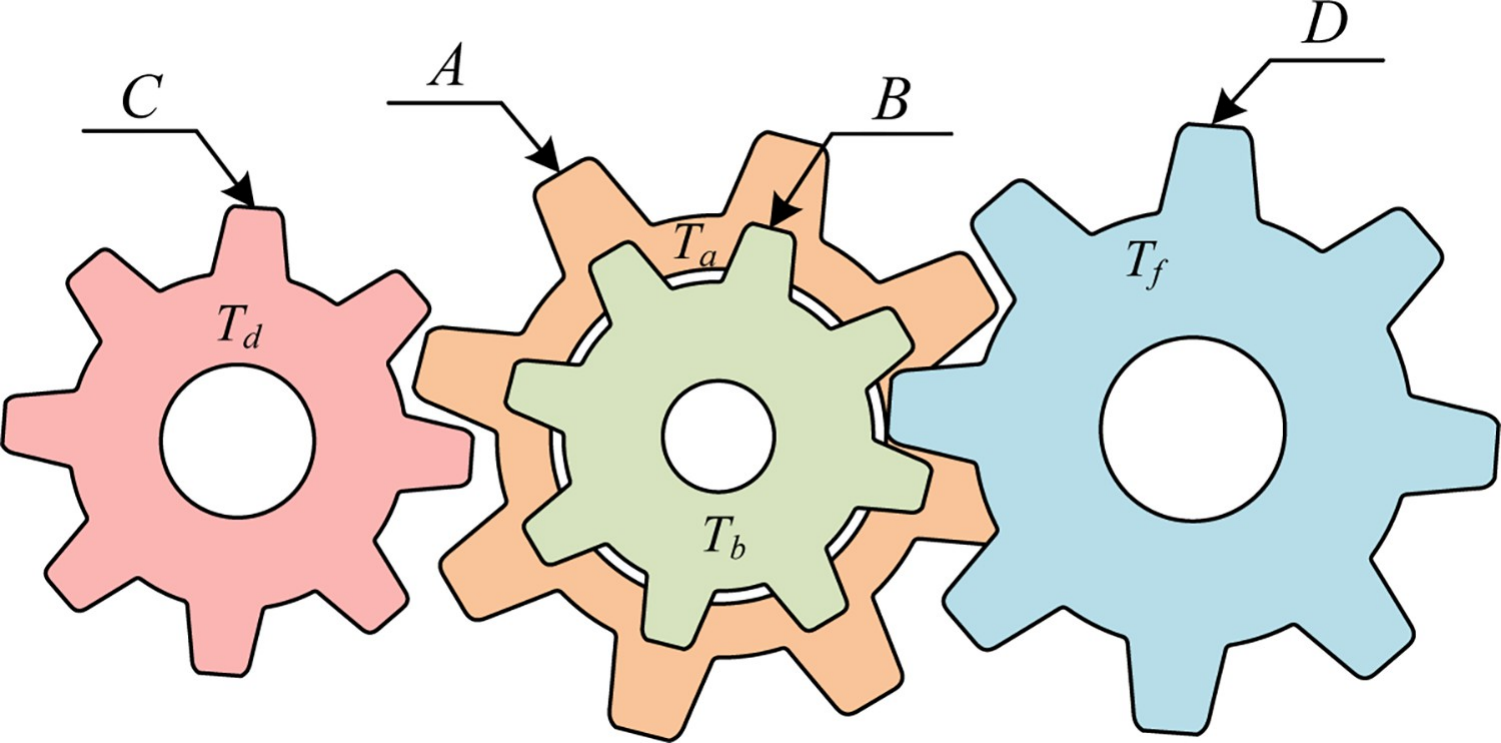
Gear train design problem.

The optimization objective of the gear train design problem is to minimize the gear transmission ratio. The corresponding topological diagram for this problem is shown in Fig 15 [8]. In this context, *T*_*a*_, *T*_*b*_, *T*_*d*_, and *T*_*f*_ represent the number of teeth on four distinct gears, serving as the four significant decision variables that determine the gear transmission ratio. The gear ratio is given as *T*_*b*_/*T*_*a*_⋅*T*_*d*_/*T*_*f*_.

Let $\vec{x}=[x_{1},x_{2},x_{3},x_{4}]=[T_{a},T_{b},T_{d},T_{f}]$, $f(\vec{x})$ represents the optimal performance of the transmission ratio. Then the mathematical model of the gear train design problem is described as:

$$\min\;f(\vec{x})={\left(\frac{1}{6.931}-\frac{x_{3}x_{2}}{x_{1}x_{4}}\right)}^{2}$$ (26)

Where *x*_1_∈[12,60], *x*_2_∈[12,60], *x*_3_∈[12,60], *x*_4_∈[12,60].

Table 9 presents the optimization results of the gear train design problem using six metaheuristic algorithms, including MSHHOTSA. According to the table, both MSHHOTSA and HHO have achieved the optimal gear transmission ratio with an objective function value of $f(\vec{x})=0.00E+00$. However, the optimal design parameters obtained by these two algorithms differ. MSHHOTSA identifies the optimal design parameter as $\vec{x}=[35.5638,21.2577,13.7114,56.8048]$, whereas HHO identifies $\vec{x}=[53.3478,17.2429,16.6616,37.3257]$. This discrepancy implies that various gear combinations can lead to the best gear transmission ratio. Consequently, in practical engineering problems, it becomes essential not only to consider optimizing the transmission ratio but also to select an appropriate gear combination based on the actual cost of each gear.

**Table 9:** Optimal results of the different algorithms on the gear train design problem.

Algorithm	*T*_*a*_	*T*_*b*_	*T*_*d*_	*T*_*f*_	$f(\vec{x})$
BOA	51.9365	24.4399	12.0000	39.1382	4.43E-13
GWO	55.1753	13.3309	35.8167	59.9787	3.53E-18
MVO	55.6405	24.0358	15.7152	47.0526	1.07E-15
HHO	53.3478	17.2429	16.6616	37.3257	0.00E+00
TSA	54.0750	18.1623	20.5854	47.9214	5.82E-16
MSHHOTSA	35.5638	21.2577	13.7114	56.8048	**0.00E+00**

### 5.4 Speed reducer design problem

**Fig 16:**
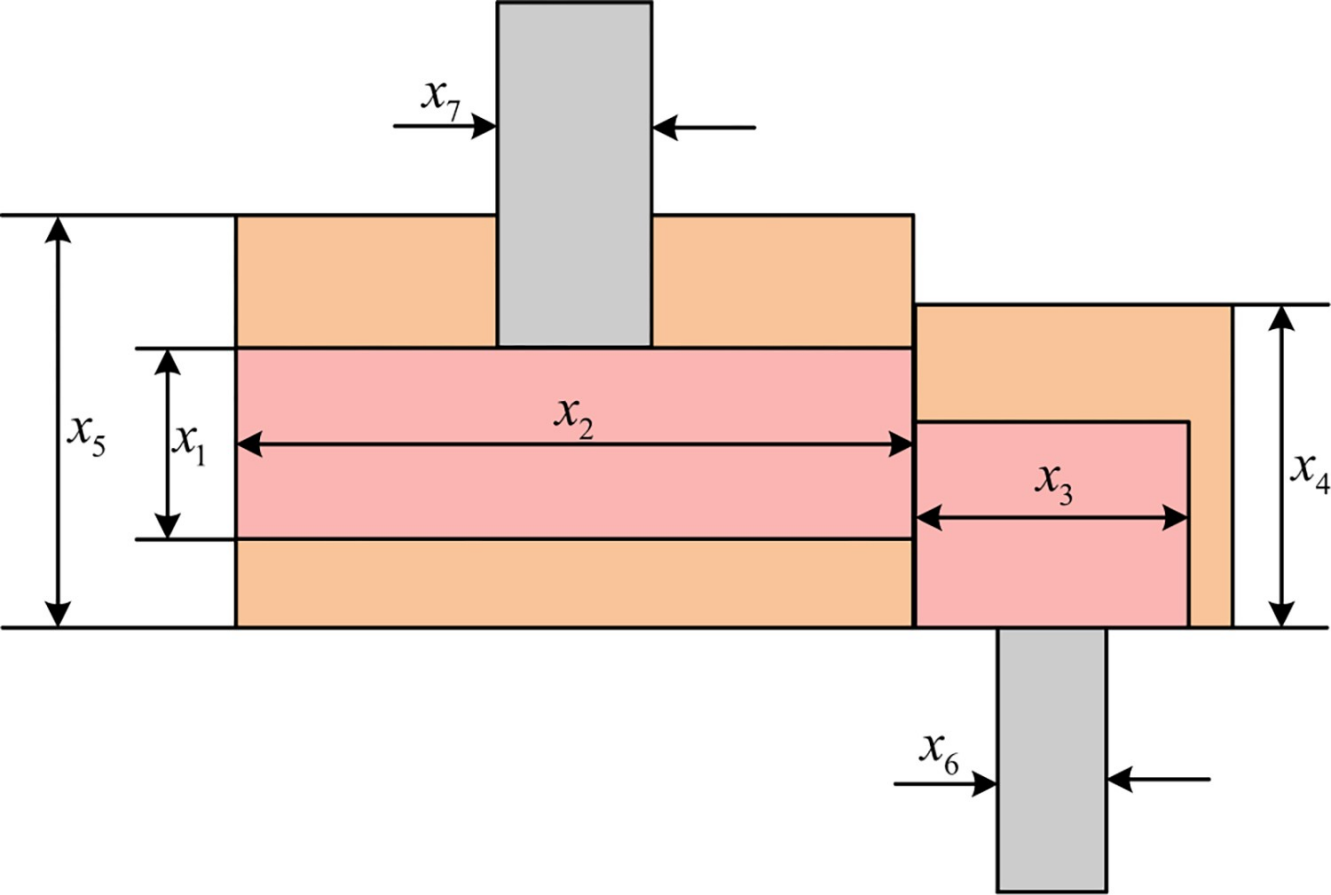
Speed reducer design problem.

The primary optimization objective of the speed reducer design problem is the minimization of the total weight of the speed reducer. The corresponding topological diagram depicting the system’s configuration is presented in Fig 16 [72]. In this particular problem, seven essential decision variables play a crucial role in determining the overall weight of the speed reducer. These variables encompass the gear face width, denoted as *b*, the gear module, represented by *m*, the number of teeth on the pinion gear, denoted as *p*, the length of the first shaft between bearings, designated as *l*_1_, the length of the second shaft between bearings, denoted as *l*_2_, the diameter of the first shaft, specified as *d*_1_, and the diameter of the second shaft, identified as *d*_2_. Collectively, these variables are succinctly represented as the vector $\vec{x}=[x_{1},x_{2},x_{3},x_{4},x_{5},x_{6},x_{7}]=[b,m,p,l_{1},l_{2},d_{1},d_{2}]$. Moreover, the function $f(\vec{x})$ signifies the total weight of the speed reducer, thereby encapsulating the overall essence of the design optimization. The mathematical model that governs the speed reducer design problem can be explicitly expressed through Eqs (27) and (28).

$$\begin{array}{l} \min\;f(\vec{x})=0.7854x_{1}x_{2}^{2}(3.3333x_{3}^{2}+14.9334x_{3}-43.0934)\\ \;-1.508x_{1}(x_{6}^{2}+x_{7}^{2})+7.4777(x_{6}^{3}+x_{7}^{3})+0.7854(x_{4}x_{6}^{2}+x_{5}x_{7}^{2}) \end{array}$$ (27)

$$s.t.\{\begin{array}{l} g_{1}(\vec{x})=\frac{27}{x_{1}x_{2}^{2}x_{3}}-1\leq 0\\ g_{2}(\vec{x})=\frac{397.5}{x_{1}x_{2}^{2}x_{3}^{2}}-1\leq 0\\ g_{3}(\vec{x})=\frac{1.93x_{4}^{3}}{x_{2}x_{3}x_{6}^{4}}-1\leq 0\\ g_{4}(\vec{x})=\frac{1.93x_{5}^{3}}{x_{2}x_{3}x_{7}^{4}}-1\leq 0\\ g_{5}(\vec{x})=\frac{\sqrt{{\left(745x_{4}/x_{2}x_{3}\right)}^{2}+16.9\times 10^{6}}}{110.0x_{6}^{3}}-1\leq 0 \end{array}\{\begin{array}{l} g_{6}(\vec{x})=\frac{\sqrt{{\left(745x_{5}/x_{2}x_{3}\right)}^{2}+157.5\times 10^{6}}}{85.0x_{6}^{3}}-1\leq 0\\ g_{7}(\vec{x})=\frac{x_{2}x_{3}}{40}-1\leq 0\\ g_{8}(\vec{x})=\frac{5x_{2}}{x_{1}}-1\leq 0\\ g_{9}(\vec{x})=\frac{x_{1}}{12x_{2}}-1\leq 0\\ g_{10}(\vec{x})=\frac{1.5x_{6}+1.9}{x_{4}}-1\leq 0\\ g_{11}(\vec{x})=\frac{1.1x_{7}+1.9}{x_{5}}-1\leq 0 \end{array}$$ (28)

Where *x*_1_∈[2.6,3.6], *x*_2_∈[0.7,0.8], *x*_3_∈[17,28], *x*_4_∈[7.3,8.3]. *x*_5_∈[7.8,8.3], *x*_6_∈[2.9,3.9], *x*_7_∈[5.0,5.5].

Table 10 presents the optimal solutions obtained through the utilization of six metaheuristic algorithms, which include MSHHOTSA, to address the speed reducer design problem. As per the data showcased in Table 10, within the confines of the same experimental conditions, the MSHHOTSA algorithm has successfully attained the optimal design solution, leading to the minimization of the total weight of the speed reducer. The associated optimal design parameters are denoted as $\vec{x}=[3.5,0.7,17,7.3,7.7153,3.3502,5.2867]$, and the corresponding optimal objective function value stands at $f(\vec{x})=2994.4711$. This significant achievement points towards the MSHHOTSA algorithm’s remarkable optimization capability when it comes to addressing the speed reducer design problem, underscoring its superior effectiveness in identifying the optimal solution.

**Table 10:** Optimal results of the different algorithms on the speed design reducer problem.

Algorithm	*b*	*m*	*p*	*l*_1_	*l*_2_	*d*_1_	*d*_2_	$f(\vec{x})$
BOA	3.5420	0.7099	17.0952	7.7471	7.8751	3.8716	5.2801	1.87E+10
GWO	3.5027	0.7000	17.0000	7.3695	7.7790	3.3509	5.2868	2997.8368
MVO	3.5008	0.7000	17.0000	7.4931	7.9720	3.3715	5.2874	3008.0690
HHO	3.5008	0.7000	17.0000	7.3307	7.7936	3.3671	5.2867	3001.1165
TSA	3.5194	0.7000	17.0000	7.5551	7.8778	3.3844	5.2903	3019.0513
MSHHOTSA	3.5000	0.7000	17.0000	7.3000	7.7153	3.3502	5.2867	**2994.4711**

### 5.5 Parameters optimization of proportion integral derivative controller

**Fig 17:**
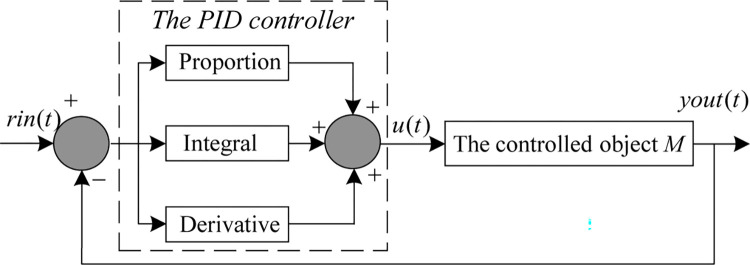
The proportion integral derivative controller.

In general, a PID controller consists of three main units: the proportional unit (P), the integral unit (I), and the differential unit (D). Its primary objective is to devise a control strategy based on the deviation between the actual value of the controlled object and the desired value. Subsequently, the controller stabilizes the closed-loop control system through parameter optimization, thereby achieving the ultimate control objective [3, 15]. Fig 17 illustrates the execution principle of the PID controller.

In the figure, *rin*(*t*) is the system input signal, *yout*(*t*) is the system output signal, and *e*(*t*) is the system error. From Fig 16, the transfer function of the PID controller is expressed as:

$$u(t)=K_{p}\cdot e(t)+K_{i}\cdot \int_{0}^{1}e(t)+K_{d}\cdot \frac{d(e(t))}{dt}$$ (29)

Where *K*_*p*_ is the proportion factor, *K*_*i*_ is the integration factor, *K*_*d*_ is the derivative factor, and *e*(*t*) is the systematic error.

To verify the optimization performance of MSHHOTSA for PID parameters, the second-order delay system in the literature [3] was used as a simulation example and numerical experiments were conducted.

$$G(s)=\frac{50}{4.23s^{2}+19.1801s+1}$$ (30)

where *G*(*s*) is the transfer function and *s* represents the continuous system. Then the objective function is expressed as:

$$F_{P}=\int_{0}^{\infty }(\omega_{1}|e(t)|+\omega_{2}u^{2}(t))+\omega_{3}|e(t)|dt$$ (31)

where *e*(*t*)<0 represents the error between the system input and output; *u*(*t*) represents the system output signal, *ω*_3_|*e*(*t*)| is the overshoot item; *ω*_1_, *ω*_2_, *ω*_3_ is the weight value, the range is between [0, 1], and *ω*_3_>>*ω*_1_.

**Fig 18:**
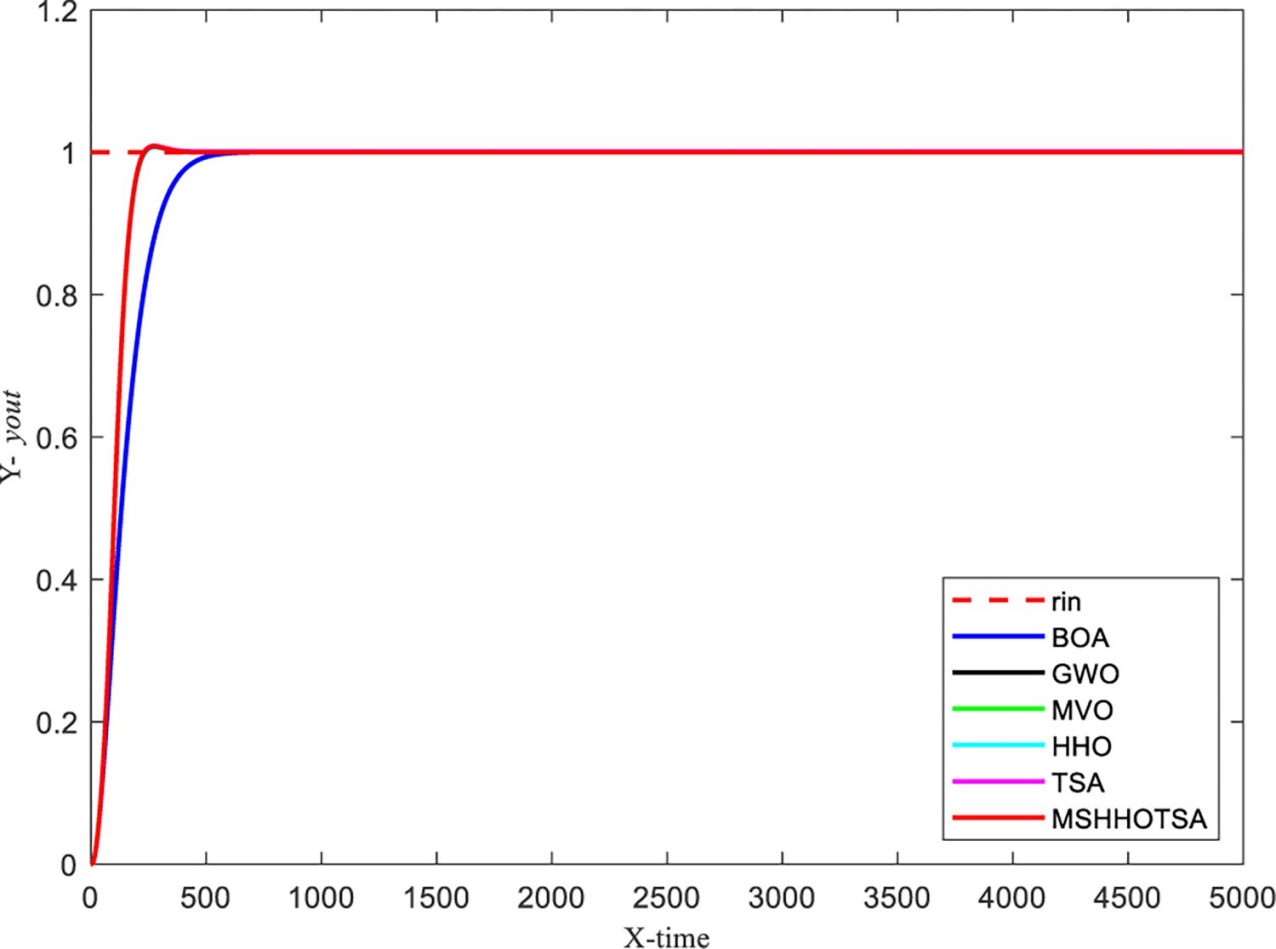
Curve of step response signal of 6 algorithms.

**Fig 19:**
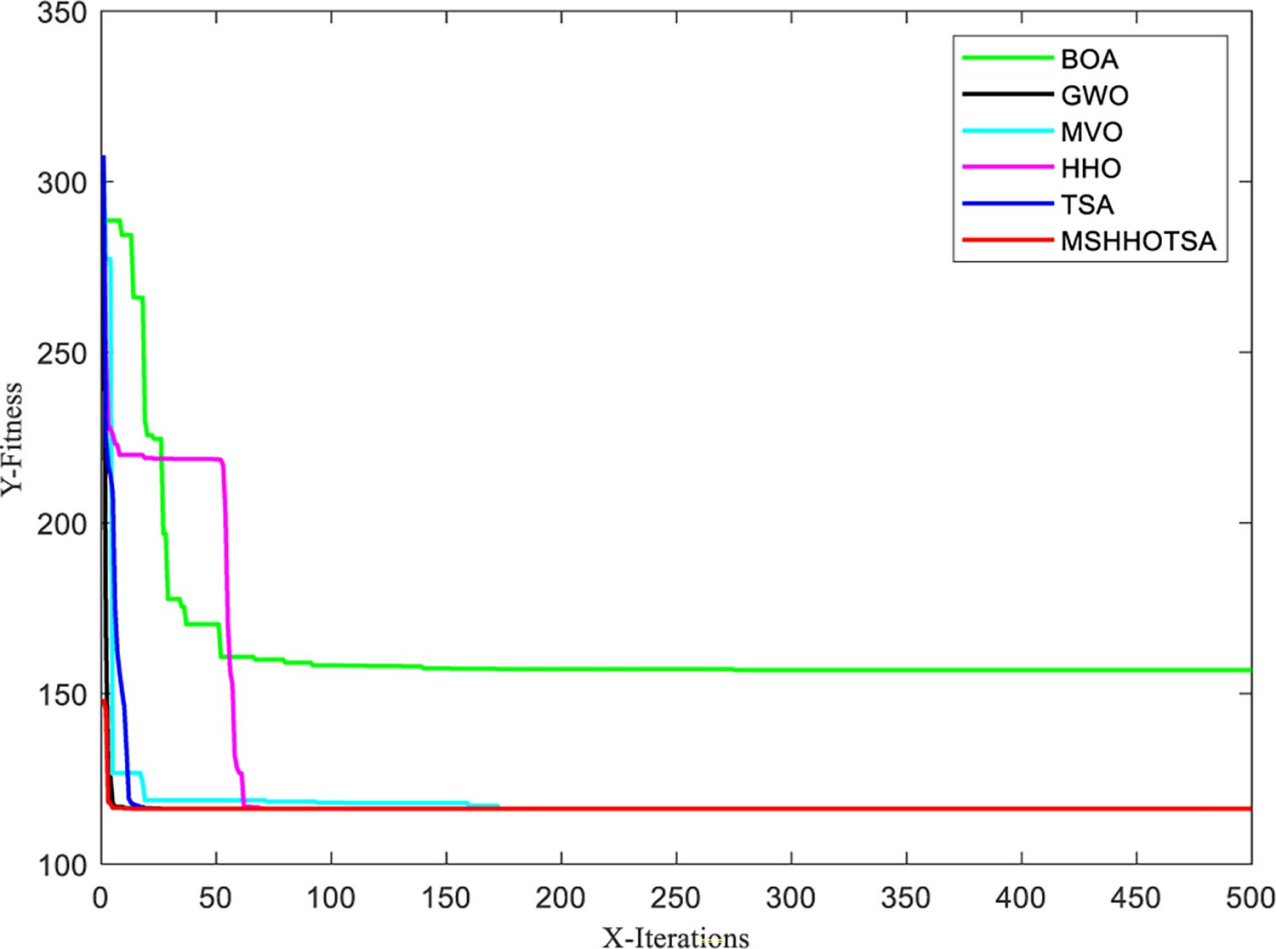
Convergence curves for each algorithm to solve pressure vessel design problem.

To optimize the PID controller, we set the initial population size of the algorithm to 30, and the maximum number of iterations is 500. The input signal is a unit step signal, and the sampling time is 0.001s. Additionally, the search range for the PID parameters *K*_*p*_, *K*_*i*_, and *K*_*d*_ is defined as [0,50]. With these settings, we obtained the parameter optimization results for each algorithm, as illustrated in Figs 18 and 19.

From the iterative convergence curves of the six algorithms, it is evident that MSHHOTSA exhibits faster convergence compared to the other five comparative algorithms. This observation signifies that MSHHOTSA demonstrates superior convergence performance in finding the best solution. Additionally, the step response signal output curves in Fig 18 show that MSHHOTSA has smaller overshoot and adjustment time when compared to the comparison algorithms. This further supports the conclusion that MSHHOTSA outperforms the comparison algorithms in terms of system stability.

### 5.6 Discussion

In this section, we conducted tests on five practical engineering applications to evaluate the optimization performance of the MSHHOTSA algorithm and obtained experimental results. These experimental results demonstrate that MSHHOTSA achieved the best optimization for all five practical engineering applications under the same experimental conditions. This further validates the stronger practicality and superiority of MSHHOTSA in addressing real-world engineering optimization problems, showcasing its robustness.

The experimental results strongly support the successful application of the MSHHOTSA algorithm in practical engineering scenarios. However, it was observed during the experiments that excellent initial values remain crucial in ensuring the rapid convergence of the MSHHOTSA algorithm. For specific engineering optimization problems, MSHHOTSA tends to gradually approach the optimal solution from the boundary conditions, resulting in slower convergence speed in the early iterations. Different combinations of parameters in various metaheuristic algorithms may lead to the same optimal results when solving real-world engineering problems.

Therefore, when using the MSHHOTSA algorithm to solve engineering optimization problems, it is beneficial to employ some simple methods to obtain initial values for the problem. This approach can effectively improve the convergence performance of the MSHHOTSA algorithm. Additionally, when different parameter combinations yield the same optimal results in practical engineering applications, it is essential to introduce additional constraints based on the specific problem to select the most suitable algorithm.

In conclusion, despite limitations, the MSHHOTSA algorithm’s outstanding performance in solving the five engineering optimization problems offers valuable insights for enhancing and optimizing other metaheuristic algorithms. In practical applications, we can leverage the strengths of the MSHHOTSA algorithm and combine them with other improvement measures to further enhance its performance and applicability.

## 6. Conclusion and future works

This paper proposes a novel hybrid algorithm called “Multi-Strategy Tunicate Swarm Algorithm with Hybrid Harris Optimization” (MSHHOTSA). The inspiration for this method comes from observing the positional movements of tunicate swarms and Harris hawks during their cooperative hunting in nature. Also, we drew inspiration from the neighborhood and heat map distribution concepts. In this study, we employed various improvement strategies. These strategies involved hyperbolic tangent domain modification for individual positions within the tunicate swarm, updating the non-linear convergence factor to influence the collective behavior of the swarm, and integrating a hybrid Harris Hawks optimization algorithm to update the population’s positions. To evaluate the optimization performance of MSHHOTSA, we used eight standard benchmark functions and the CEC2019 benchmark functions and compared it against seven well-known metaheuristic benchmark functions. Additionally, we tested the applicability of MSHHOTSA on five real-world engineering problems and conducted comprehensive evaluations by comparing its performance against five other metaheuristic algorithms.

MSHHOTSA exhibits superior optimization performance when solving benchmark functions under the same experimental conditions, as indicated by smaller means, standard deviations, and higher overall rankings. Moreover, the convergence curves of MSHHOTSA for 18 benchmark functions further validate its ability to escape local optima and display improved convergence performance during the iterative process. The algorithm’s runtime results also confirm the faster convergence speed of MSHHOTSA. The statistical significance of MSHHOTSA has been verified through the Wilcoxon rank-sum test. Finally, the application results on five real-world engineering problems demonstrate MSHHOTSA’s superior convergence accuracy and global optimization ability. Compared to other experimental algorithms in this paper, MSHHOTSA showcases higher competitiveness.

In the future, we intend to develop specific single-objective optimization frameworks using the improved algorithm and utilize them to address challenges in feature selection and image segmentation. Moreover, we will strive to introduce novel multi-objective optimization algorithms based on the enhanced algorithm to tackle complex multi-objective optimization problems. These investigations will significantly broaden the application scope of our algorithm across various domains while also elevating its overall effectiveness and performance.

## Supporting information

S1 Data (ZIP)
